# The Antimicrobial, Antibiofilm and Anti-Inflammatory Activities of P13#1, a Cathelicidin-like Achiral Peptoid

**DOI:** 10.3390/ph16101386

**Published:** 2023-09-30

**Authors:** Valeria Cafaro, Andrea Bosso, Ilaria Di Nardo, Assunta D’Amato, Irene Izzo, Francesco De Riccardis, Marialuisa Siepi, Rosanna Culurciello, Nunzia D’Urzo, Emiliano Chiarot, Antonina Torre, Elio Pizzo, Marcello Merola, Eugenio Notomista

**Affiliations:** 1 Department of Biology, University of Naples Federico II, 80126 Naples, Italy; vcafaro@unina.it (V.C.); andrea.bosso@unina.it (A.B.); ilaria.dinardo@unina.it (I.D.N.); marialuisa.siepi@unina.it (M.S.); rosanna.culurciello@unina.it (R.C.); durzonunzia@gmail.com (N.D.); eliodoro.pizzo@unina.it (E.P.); 2 Department of Chemistry and Biology “A. Zambelli”, University of Salerno, 84084 Fisciano, Italy; asdamato@unisa.it (A.D.); iizzo@unisa.it (I.I.); dericca@unisa.it (F.D.R.); 3 GSK, 53100 Siena, Italy; emiliano.x.chiarot@gsk.com (E.C.); torreantonina@gmail.com (A.T.)

**Keywords:** antimicrobial peptide, peptidomimetic, peptoid, ESKAPE pathogens, multidrug resistance, biofilm

## Abstract

Cationic antimicrobial peptides (CAMPs) are powerful molecules with antimicrobial, antibiofilm and endotoxin-scavenging activities. These properties make CAMPs very attractive drugs in the face of the rapid increase in multidrug-resistant (MDR) pathogens, but they are limited by their susceptibility to proteolytic degradation. An intriguing solution to this issue could be the development of functional mimics of CAMPs with structures that enable the evasion of proteases. Peptoids (*N*-substituted glycine oligomers) are an important class of peptidomimetics with interesting benefits: easy synthetic access, intrinsic proteolytic stability and promising bioactivities. Here, we report the characterization of P13#1, a 13-residue peptoid specifically designed to mimic cathelicidins, the best-known and most widespread family of CAMPs. P13#1 showed all the biological activities typically associated with cathelicidins: bactericidal activity over a wide spectrum of strains, including several ESKAPE pathogens; the ability to act in combination with different classes of conventional antibiotics; antibiofilm activity against preformed biofilms of *Pseudomonas aeruginosa*, comparable to that of human cathelicidin LL-37; limited toxicity; and an ability to inhibit LPS-induced proinflammatory effects which is comparable to that of “the last resource” antibiotic colistin. We further studied the interaction of P13#1 with SDS, LPSs and bacterial cells by using a fluorescent version of P13#1. Finally, in a subcutaneous infection mouse model, it showed antimicrobial and anti-inflammatory activities comparable to ampicillin and gentamicin without apparent toxicity. The collected data indicate that P13#1 is an excellent candidate for the formulation of new antimicrobial therapies.

## 1. Introduction

The diffusion of multi-drug resistant bacteria has focused attention on the need for new antimicrobials. Cationic antimicrobial peptides (CAMPs), which are essential components of the innate immune system in eukaryotes, are small peptides with broad-spectrum antimicrobial activity [1,2]. They are very promising antimicrobials for two main reasons: (a) CAMP-resistant strains have rarely been isolated [3,4,5,6,7], and (b) activity has been observed on both actively dividing and resting cells (e.g., the subpopulations of bacterial biofilms) [8], differing from several conventional antibiotics.

The peculiarities of CAMPs derive from their unusual mechanism of action and target [1,2]. Indeed, unlike conventional antibiotics, the main targets of CAMPs are cell membranes (inner and/or outer). CAMPs share an abundance of basic and hydrophobic residues and the ability to adopt an amphipathic conformation upon interacting with membranes. They are usually unstructured in aqueous solutions, but their positive net charge drives the initial absorption to bacterial membranes which are rich in negative phospholipids, and their amphipathic structure allows for their insertion into the membrane. CAMPs alter the fluidity, thickness and curvature of the membrane; moreover, they can induce the formation of pores, which are lethal both to actively dividing and resting cells, or even cause the micellization of the membrane. Furthermore, several CAMPs show antibiofilm activity, being able to prevent the adhesion of bacterial cells to surfaces, kill cells inside the biofilm matrix or even induce the disaggregation of the biofilm by interacting with the components of the extracellular matrix [9,10,11]. The multi-target mechanism of CAMPs makes the development of bacterial resistance, as has been observed for conventional antibiotics, very difficult and unlikely.

In addition to directly killing bacteria, several CAMPs also display immunomodulatory functions, including the ability to inhibit the endotoxin-induced production of proinflammatory cytokines [12]. In most cases, this modulatory activity is due to direct binding to endotoxins like lipopolysaccharides (LPSs), and lipoteichoic acid (LTA) [13,14,15,16,17,18,19,20]. Thus, CAMPs act as scavengers, segregating endotoxins in complexes which cannot bind to their receptors (e.g., the Toll-like receptors TLR-2 and TLR-4). Very intriguingly, the cationic/amphipathic nature of CAMPs seems to also be responsible for their ability to bind to endotoxins and endotoxin receptors [21].

This peculiar combination of properties makes CAMPs very attractive drugs. However, their sensitivity to proteolytic degradation can reduce their half-lives and, most importantly, limits their possible routes of administration. In this regard, it is particularly interesting to note that polymyxins, natural CAMPs that are widely used as topical and systemic antibiotics [22], contain several modified amino acids, protected chain termini and a cyclic structure which confer resistance to proteolysis.

In order to fully exploit the pharmacological potential of CAMPs, an intriguing strategy is to develop antimicrobial peptidomimetics which are able to structurally and functionally mimic CAMPs but are resistant to proteases.

Several peptidomimetic have been used to develop CAMP mimics [23], and peptoids are among the most promising [24]. Peptoids, oligomers of *N*-substituted glycines, are regioisomers of peptides characterized by the fact that the side chains are shifted from the α-carbon to the nitrogen atom of the polyamide backbone. This feature makes peptoids resistant to endogenous proteases [25]. Moreover, the straightforward synthesis of peptoids (based on the solid-phase “sub-monomer” approach in which no protective groups are required) expedites their preparation (in linear and cyclic forms) [24] and justifies their use as anti-infective agents. In peptoids, the presence of substituents on the nitrogen atom of the amide backbone hampers the formation of α-helices or strands, which are typically present in peptides. Additionally, peptoids can form polyproline helices I and II so that structured amphipathic frames can be designed and modelled in silico via molecular dynamics or Monte Carlo methods [26].

Most designed antimicrobial peptoids were inspired by helical CAMPs which, in the presence of lipids, can adopt an amphipathic helical structure responsible for their strong interaction with bacterial membranes [27]. As the peptoid backbone is devoid of stereogenic centers, peptoids can form helical structures with defined helicity only if they contain a large proportion of chiral aromatic side chains, such as those present in *N*-(*S*)- and *N*-(*R*)-(1-phenylethyl)-glycine (*N*spe and *N*rpe) or similar residues. In fact, most designed peptoids were based on a three-residue repeat, (B-A-A)_n_, in which B is a basic residue and A is a hydrophobic residue, usually a chiral aromatic residue, e.g., *N*spe or *N*rpe [27,28,29,30]. Peptoids containing only *N*spe residues fold as right-handed helices similar to polyproline I helix (with all the peptide bonds in the *cis* configuration and three residues per turn), whereas peptoids containing only *N*rpe fold as left-handed helices [27,31]. However, more recent studies have demonstrated that helicity and defined chirality are not essential prerequisites for a high level of antimicrobial activity, while a reduced degree of helicity decreases toxicity, thus improving the pharmacological index [32]. These findings are not completely unexpected as many highly active CAMPs are not helical and some have no regular secondary structure, even when bound to membranes (e.g., indolicidin and tritrpticin [33]). Moreover, the stability of several CAMPs was improved by changing one or more L-amino acids into a D-amino acid [13,34,35]. Even if these changes often significantly alter the secondary structure, antimicrobial activity is generally retained or even improved [34,35,36,37].

Here, we describe the biological properties of P13#1, a designed 13-residue peptoid comprising only achiral monomers, with broad-spectrum bactericidal activity and an LPS-scavenging activity comparable to that of polymyxin E (also known as colistin), a lipopeptide with strong and specific binding to LPSs [22]. Moreover, an antibiofilm activity greater than that of LL-37, the only human cathelicidin [38], was observed for P13#1. Finally, P13#1 was highly effective in vivo in a mouse model of subcutaneous infection in which it showed neglectable toxicity.

## 2. Results and Discussion

### 2.1. The Design of Peptoid P13#1

Peptoid P13#1 was designed taking into consideration our previous work on the identification of cryptic CAMP-like sequences [39]. This method has allowed for the identification of many human peptides [40,41,42,43] and, more recently, the identification of dozens of new CAMPs via screening the whole human proteome [44]. We showed that a correlation does exist between the antimicrobial potency (defined as log(1000/MIC)) of a peptide and a score dependent on the net charge and hydrophobicity of the peptide and two strain-dependent constants. The relationship is linear for scores between about 6 and 10–11, whereas for scores higher than 11, an increase in the score does not necessarily increase the potency of the peptide. Even if the “ideal” composition of a CAMP depends on a specific bacterial strain, our analysis suggested that on the average, a high-potency CAMP should contain about 40% basic residues (Lys and Arg) and 60% of the most hydrophobic residues (Trp, Phe and Leu). In particular, a peptide with six Lys/Arg residues and seven Trp/Phe/Leu residues would have a score between 11 and 13, depending on the specific set of parameters used for the calculation (the hydrophobicity score list and strain-dependent constants), thus being the shortest peptide with score higher than the linearity window.

Therefore, to verify whether data acquired from the analysis of CAMPs can be translated to peptoids as well, we decided to synthesize a peptoid with six basic and seven aromatic residues. As a basic residue we preferred NLys, the peptoid analogue of lysine, over *N*Arg because the use of an *N*Lys residue facilitates the synthetic process; moreover, according to our analysis, in high-scoring peptides, Lys and Arg are equivalent. As an aromatic residue, we selected *N*-(2-phenylethyl)-glycine (*N*pet), the peptoid analogue of homophenylalanine, as this residue is slightly more hydrophobic than *N*Phe (the peptoid analogue of Phe) and is more stable and easier to handle with respect to the peptoid analogues of Trp [45].

**Figure 1:**
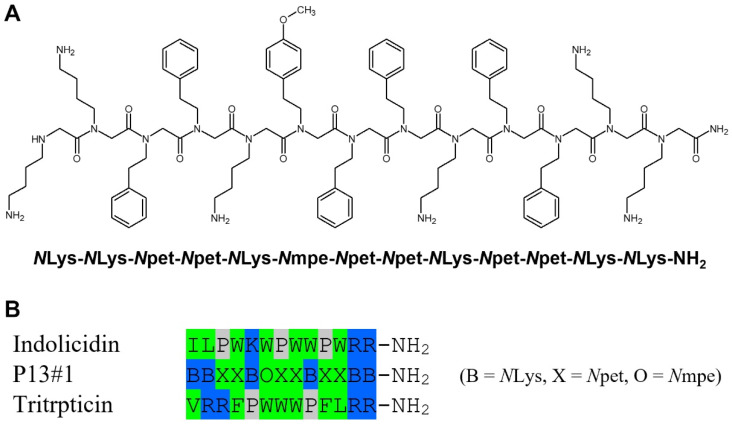
The primary structure of P13#1. (**A**) The covalent structure and sequence of P13#1. (**B**) A comparison between the sequences of P13#1, indolicidin and tritrpticin (color code: green—hydrophobic residues; blue—cationic residues; gray—prolines).

Finally, we selected a specific sequence of six *N*Lys residues and seven *N*pet residues (Figure 1A), providing an amphipathic structure both when folded as (all-*cis*) polyproline-I-like and (all-*trans*) polyproline-II-like helices, as discussed below. One of the *N*pet residues was changed to *N*-[2-(4-methoxyphenyl)ethyl]-glycine (*N*mpe) in order to obtain a peptoid with a good extinction coefficient at 280 nm, thus simplifying the purification and quantification steps. It is worth noting that the sequence of P13#1 shows some similarities with the sequences of bovine indolicidin and porcine tritrpticin, the two shortest and yet very active mammalian cathelicidins [33], even if P13#1 was not specifically designed to mimic these two CAMPs (Figure 1B).

**Figure 2:**
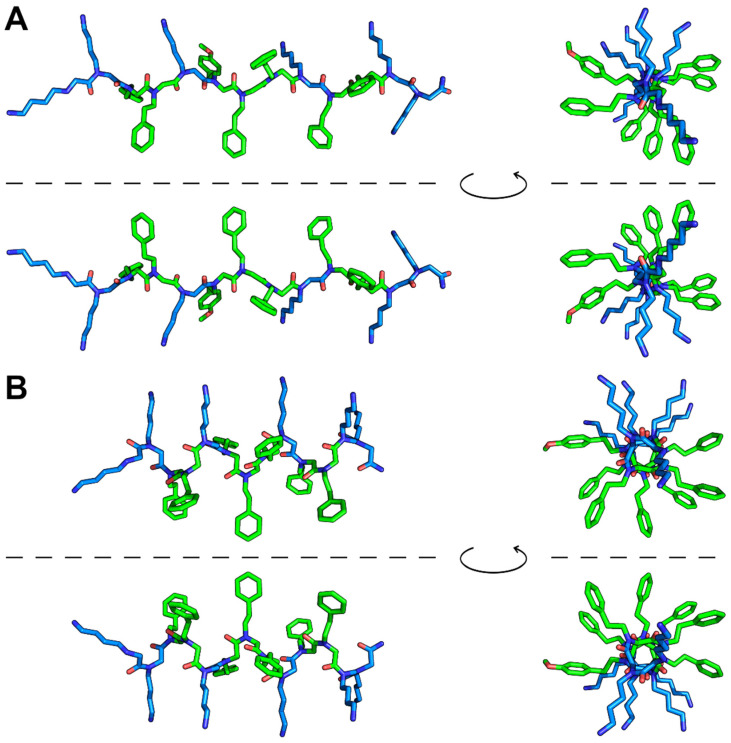
Monte Carlo minimized structures of P13#1 using octanol solvation. (**A**) All-*trans* polyproline-helix-II-like conformation. (**B**) All-*cis* polyproline-helix-I-like conformation. In both panels, the structures above and below the line are left-handed and right-handed helices, respectively. The structures on the left are shown from the side of the helix, whereas the structures on the right are shown from the N-terminus. Color code: carbon in hydrophobic residues—green; carbon in cationic residues—light blue; oxygen—red; nitrogen—dark blue. Hydrogen atoms are not shown.

Monte Carlo molecular modeling was used to perform an in silico conformational analysis. Two implicit solvation functions were used to predict the conformation of the peptoid in water and in a membrane-mimicking environment (octanol), respectively. Using octanol solvation, the more extended polyproline-helix-II-like conformation (Figure 2A) was found to be about 2.9 kcal/mole more stable than the polyproline-helix-I-like conformation (Figure 2B). Even more interestingly, long minimizations did not change the regular helical conformations, thus indicating that in a membrane-mimicking environment, the two conformations represent very distinct local minima. This is because the effective solvation of the hydrophobic side chains by octanol prevents the formation of intramolecular contacts among them which, in turn, could stabilize the more compact conformations. We want to underline that as P13#1 has no stereogenic centers, each helical conformation can exist both as isoenergetic, enantiomorphous left- and right-handed helices (Figure 2). This implies that in solvents (or any achiral environment) favoring helical conformations, left- and right-handed helices will coexist.

Different from what was observed in octanol, a minimization performed using water solvation provided several different structures with similar energies and less regular helical conformations due to the formation of intramolecular contacts among the side chains of the aromatic residues. Some of these conformations are shown in Figure S1A–D in Supplementary Materials.

We also explored the stability of conformations containing mixed *cis* and *trans* peptide bonds via a random approach described in detail in the Methods section. When octanol solvation was used, all the minimized structures showed energy values slightly higher than the energy value of the all-*trans*/fully extended polyproline-II-like conformation (Figure S2A–D in Supplementary Materials). Very interestingly, some of these conformations (e.g., those shown in panels B and C) are similar to the NMR structures of bovine indolicidin in micelles of SDS and dodecylphosphocholine (Figure S2E,F in Supplementary Materials) [46]. On the contrary, when using water solvation, we obtained several conformations with energy values slightly lower than those obtained starting from the all-*cis* and all-*trans* conformations. Also, these structures were characterized by intramolecular contacts among the hydrophobic side chains, leading to compact conformations that were able to shield the hydrophobic groups from the solvent (Figure S1E–H in Supplementary Materials).

Even if the use of implicit solvation functions does not allow for a fully quantitative analysis, our approach nonetheless shows that P13#1 is a very flexible molecule able to adopt an extended, more regular conformation in a hydrophobic environment and several more compact, less regular conformations with turn-like elements in water. This is a typical behavior of membrane-targeting CAMPs.

### 2.2. Synthesis of P13#1

The synthesis of P13#1 was performed via a solid-phase extraction procedure, using the classical “sub-monomeric” approach [47,48]. The solid support chosen was a cross-linked polystyrene, utilized with Rink’s amide linker, to obtain a C-terminal amide after its cleavage from resin. The first step was bromoacetylation using diisopropyl carbodiimide, followed by nucleophilic substitution with *tert*-butyl *N*-(4-aminobutyl)carbamate, which furnished the first monomer. By iterating the bromoacetylation reaction of the free amine terminal group and the nucleophilic displacement with the appropriate amine, we obtained the desired peptoidyl-resin. Detachment from the resin in acidic conditions and precipitation in cold diethyl ether produced the deprotected oligomer P13#1 in a yield of 68%. Interestingly, this procedure is easily scalable to obtain larger amounts of the desired substrate. In order to obtain the variant CG-P13#1 (see Section 2.6), the peptoidyl-resin was elongated, using the classical monomer approach to introduce glycine and cysteine at the N-terminus. Cleavage from the resin in acidic conditions, followed by precipitation, afforded CG-P13#1 in a yield of 19%.

### 2.3. The Antimicrobial Activity of P13#1

The Minimum Inhibitory Concentration (MIC) values of P13#1 were measured on a wide panel of Gram(+) and Gram(−) strains, including several strains belonging to the so-called ESKAPE pathogens, i.e., a relatively small group of pathogens which causes frequent hospital infections. Data are shown in Table 1.

Concerning *P. aeruginosa*, we included laboratory, highly virulent and clinical strains: PAO1 is a well-known and widely used reference strain and PA14 is a highly virulent strain [49], whereas AA2, KK27 and RP73 are clinical strains isolated from cystic fibrosis (CF) patients [50,51]. Among these, *P. aeruginosa* RP73 is a “late” strain which shows specific mutations related to its ability to cause chronic infections due to a long adaptation to the peculiar environment provided by the lung of a CF patient [51,52,53]. Regarding *S. aureus*, we included a laboratory strain (ATCC 6538P) and two clinical strains, WKZ-2, a methicillin-resistant strain [54], and Newman, a highly virulent strain [55]. As positive controls, we included three well-known antimicrobial peptides, namely, (P)GKY20, polymyxin B and vancomycin. The reference peptide (P)GKY20 is the recombinant form of GKY20, a cryptic CAMP derived from the C-terminus of human thrombin [56,57]. It is a cathelicidin-like peptide with wide-spectrum antimicrobial activity [56,57]. Polymyxin B is a cationic lipopeptide, very similar to colistin (polymyxin E), which specifically kills Gram(−) strains by binding to LPSs and inducing the permeabilization of their outer membranes [22]. Vancomycin is a cyclic glycopeptide that selectively inhibits Gram(+) strains by interfering with cell-wall synthesis. As expected for a cathelicidin mimic, P13#1 showed wide-spectrum antimicrobial activity, with MIC values generally below 2 µM. In the panel of investigated strains, the sole resistant strain was *Burkholderia cenocepacea* LMG 18863. It is worth noting that this strain was also resistant to (P)GKY20 and polymyxin B. This finding was not unexpected; in fact, it is well-known that *Burkholderia* strains are naturally resistant to membrane-targeting antimicrobial peptides due to the biosynthesis of an unusual LPS which bears positively charged groups that hinder the insertion of cationic peptides into the outer membrane, thus preventing its damage and crossing [58,59].

We also determined the Minimum Bactericidal Concentration (MBC) values of P13#1 and (P)GKY20. In both cases, MIC and MBC values were identical for all the sensitive strains; thus, as an antibacterial agent is usually regarded as bactericidal if its MBC value is no more than four times its MIC value, both (P)GKY20 and P13#1 can be fully considered bactericidal agents.

### 2.4. Peptoid–Antibiotic Interaction Study

It has been shown that the co-administration of CAMPs and antibiotics often provides synergic effects [60,61]. In particular, a recent study [61] showed that synergy is most likely to be observed when combining CAMPs with high levels of membrane-damaging activity and antibiotics with intracellular targets (e.g., DNA and protein synthesis inhibitors). It is likely that the membrane damage caused by the CAMPs could favor the penetration of antibiotics, thus enhancing their action. Based on these observations, we measured the Fractional Inhibitory Concentration Index (FIC_i_) of P13#1 on *P. aeruginosa* PAO1 and *S. aureus* ATCC 6538P and antibiotics belonging to four different classes, namely, tobramycin (an aminoglycoside), ciprofloxacin (a fluoroquinolone), meropenem (a β-lactam antibiotic) and colistin (a polymyxin) (Table 2).

Usually, an FIC_i_ < 0.5 is considered a clear indication of synergy, values between 0.5 and 1 are considered indicative of additivity, values between 1 and 2 are considered indicative of indifference and values > 2 are indicative of antagonism [62,63]. Except for meropenem, all the measured values were lower than 1, thus indicating additivity/synergy. In particular, the combination of P13#1 + colistin showed an FIC_i_ value well below 0.5, thus highlighting a strong synergistic effect. It is worth noting that synergy with colistin has also been found frequently with other CAMPs [43,60,64,65]. As polymyxins specifically damage the outer membranes of Gram(−) strains, it can be speculated that they could favor the penetration into the wall of other CAMPs which, differently from polymyxins, can also damage the inner membrane. This combined action would explain the strong synergistic effect [64]. Also, the observation that FIC_i_ values close to 0.5 were found for the P13#1 + tobramycin combination is particularly interesting for the potential pharmacological applications of P13#1. As aerosolized tobramycin and colistin are frequently used to treat airway infections (e.g., in the case of cystic fibrosis patients who develop *P. aeruginosa* infections [66]), it is possible to conceive of combination treatments consisting of these antibiotics and P13#1.

### 2.5. The Anti-Biofilm Activity of P13#1

**Figure 3:**
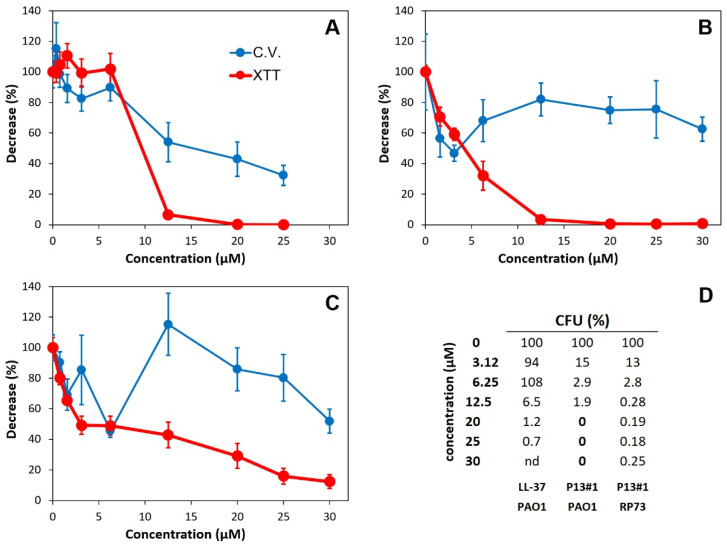
The antibiofilm activity of LL-37 (**A**) and P13#1 (**B**) on *P. aeruginosa* PAO1 and the antibiofilm activity of P13#1 on *P. aeruginosa* RP73 (**C**). Blue circles—the amount of biofilm-bound crystal violet, expressed as a percentage with respect to the untreated wells. Red circles—XTT reduction, expressed as percentage with respect to the untreated wells. (**D**) Percentage of the CFU remaining after the treatment.

Many CAMPs show the ability to inhibit biofilm formation, and the most effective are even able to eradicate preformed biofilms [67,68,69]. Accordingly, we studied the ability of P13#1 to eradicate a preformed biofilm of *P. aeruginosa* PAO1. In this case LL-37, the sole human cathelicidin, was used as a positive control because its antibiofilm activity is well known [69]. Overnight bacterial cultures were diluted to 1 × 10^8^ colony-forming units/mL (CFU/mL) in BM2 medium, and the bacterial biofilms were formed for 24 h at 37 °C in 96-well plates. The eradication of the mature biofilms was evaluated by incubating the biofilms with increasing concentrations (0–30 µM) of LL-37 or P13#1 for 4 h. The biofilm mass and cell viability were measured using crystal violet and XTT (sodium 2,3,-bis(2-methoxy-4-nitro-5-sulfophenyl)-5-[(phenylamino)-carbonyl]-2H-tetrazolium) reduction assays, respectively, and the minimum biofilm eradication concentration (MBEC) was determined by counting the viable bacterial cells inside a biofilm’s structure upon the disruption of the biofilm with 1% Triton X-100 (Figure 3).

LL-37 induced both a reduction in the biofilm’s mass and a strong reduction in cell viability at concentrations ≥ 12.5 µM (Figure 3A). These data were confirmed by determining the percentage of the CFU remaining after the treatment. At 25 µM, LL-37 caused a reduction of slightly more than 99%. However, we were not able to measure an MBEC value, which is usually defined as the concentration of an antibiofilm agent which reduces the CFU by 99.9% or more. Differing from LL-37, P13#1 did not significantly reduce the biofilm’s mass (Figure 3B) but caused a larger reduction in the cell viability, beginning from the lowest concentration assayed (1.56 µM), and a larger reduction in the CFU. Indeed, at concentrations of P13#1 ≥ 20 µM, no colony was observed, thus allowing for an MBEC = 20 µM to be defined. Therefore, it can be concluded that LL-37 kills the cells embedded into the biofilm to some extent and partially solubilizes the biofilm matrix, whereas P13#1 is not able to disrupt the biofilm’s architecture; however, it is more efficient at killing the embedded cells.

We also analyzed the antibiofilm effects of P13#1 on the clinical stain *P. aeruginosa* RP73, which is a very efficient biofilm-former (Figure 3C). The obtained results were similar to those observed on PAO1; however, it was not possible to define an MBEC value since even at the highest concentrations tested, we still found about 0.2–0.3% of the CFU. It could be speculated that the *P. aeruginosa* RP73 biofilm contains a subpopulation of cells which were not accessible or not sensitive to the peptoid. This hypothesis would be consistent with the origin of *P. aeruginosa* RP73, a late clinical strain of *P. aeruginosa* adapted to cause chronic infections.

### 2.6. Interactions of P13#1 with SDS, LPSs and Bacterial Cells

We recently developed a peptide-labelling strategy based on an environment-dependent luciferin-like fluorophore [70]. The amino and hydroxy versions of this fluorophore are strongly sensitive to environmental polarity and proton acceptor availability and/or local pH, respectively. This labelling strategy allows one to study the interactions of peptides with detergents, lipids, micelles, liposomes and even whole cells. For this reason, we designed CG-P13#1, a P13#1 variant with two additional residues at the N-terminus: cysteine and glycine. The N-terminal cysteine was needed to perform the cyclization reaction that generates the fluorophore, whereas the glycine residue was added to provide a flexible joint between the fluorophore and the peptoid (Figure S3A in Supplementary Materials). CG-P13#1 was labeled to obtain Luc-P13#1 and aLuc-P13#1, carrying the hydroxy and the amino versions of the fluorophore, respectively (Figure S3A in Supplementary Materials). Hence, we studied the interactions of the labeled P13#1 variants with sodium dodecyl sulfate (SDS) micelles, LPSs (free and micellar) and whole bacterial cells. The previously characterized labeled variants of GKY20, Luc- and aLuc-GKY20 [70], were used as controls. The excitation and emission spectra of Luc- and aLuc-P13#1 in the presence of SDS, LPSs and bacterial cells (*E. coli* ATCC 25922, *P. aeruginosa* PAO1 and *S. aureus* ATCC 6538P) were very similar to those of Luc- and aLuc-GKY20, respectively, thus demonstrating that the binding properties of GKY20 and P13#1 are comparable.

**Figure 4:**
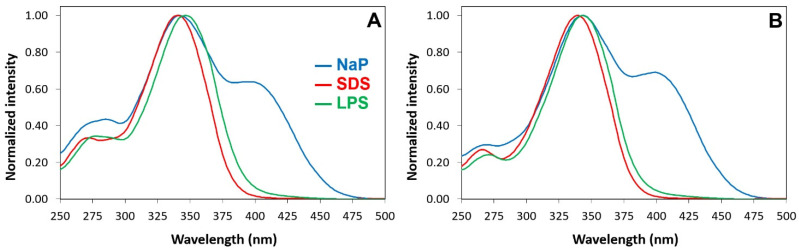
Excitation spectra of Luc-GKY20 and Luc-P13#1 in the presence of SDS or LPS micelles. (**A**) Excitation spectra of Luc-GKY20. (**B**) Excitation spectra of Luc-P13#1. Blue line—spectra recorded in sodium phosphate (NaP); red line—spectra recorded in the presence of SDS; green line—spectra recorded in the presence of LPS.

**Figure 5:**
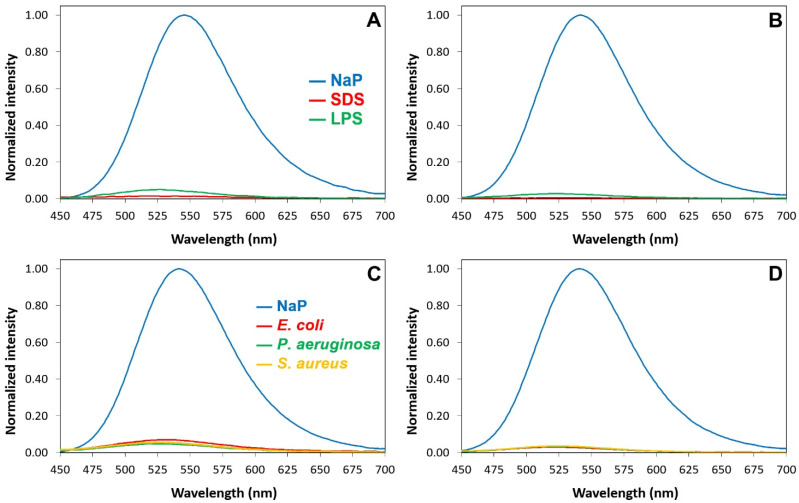
Emission spectra after excitation at 430 nm of Luc-GKY20 and Luc-P13#1 in the presence of SDS, micellar LPSs or bacterial cells. (**A**,**B**) Emission spectra of Luc-GKY20 and Luc-P13#1, respectively, in NaP (blue line), SDS (red line) and LPS (green line). (**C**,**D**) Emission spectra of Luc-GKY20 and Luc-P13#1, respectively, in NaP (blue line) or in the presence of *E. coli* (red line) *P. aeruginosa* (green line) and *S. aureus* (yellow line) cells. In each panel, the emissions in the presence of SDS, LPS and bacterial cells were normalized to the emission in NaP.

The excitation spectra in the water buffer of the Luc-labeled species show a main peak at 330 nm due to the neutral (phenolic) form of luciferin and a remarkable shoulder at 400 nm due to the phenolate form (Figure S3B in Supplementary Materials and Figure 4). The shoulder disappears when Luc-P13#1 and Luc-GKY20 bind to SDS or LPSs because binding suppresses the ionization of the phenol group (Figure S3B in Supplementary Materials). The suppression of luciferin ionization is also confirmed via the emission spectra after excitation at 430 nm, a wavelength that allows for the selective excitation of the phenolate form. The presence of SDS, LPSs or bacterial cells causes an almost complete disappearance of the fluorescence emission at 540 nm (Figure 5).

**Figure 6:**
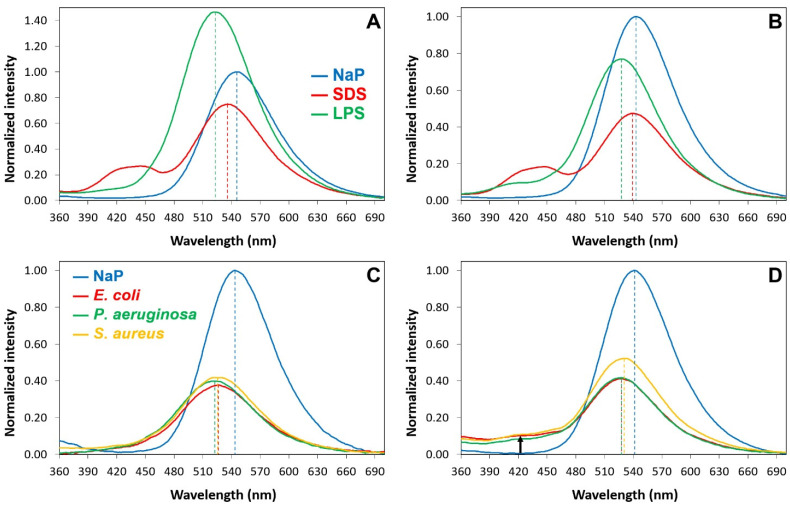
Emission spectra after excitation at 330 nm of Luc-GKY20 and Luc-P13#1 in the presence of SDS, LPSs or bacterial cells. (**A**,**B**) Emission spectra of Luc-GKY20 and Luc-P13#1, respectively, in NaP (blue line), SDS (red line) and LPSs (green line). (**C**,**D**) Emission spectra of Luc-GKY20 and Luc-P13#1, respectively, in NaP (blue line) or in the presence of *E. coli* (red line) *P. aeruginosa* (green line) and *S. aureus* (yellow line) cells. In each panel, the emissions in the presence of SDS, LPSs and bacterial cells were normalized to the emission in NaP.

The interpretation of the emission spectra after excitation of the neutral form at 330 nm (Figure S3 in Supplementary Materials) is more complicated. In an aqueous buffer, Luc-GKY20 and Luc-P13#1 show only the emission at 540 nm caused by the phenolate form (Figure 6).

**Figure 7:**
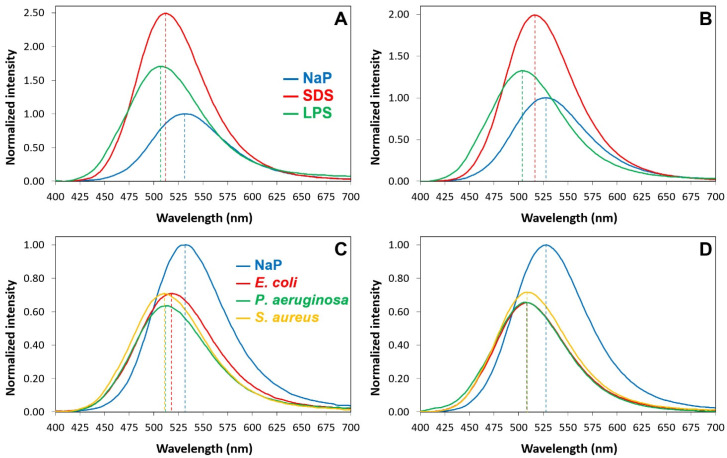
Emission spectra of aLuc-GKY20 and aLuc-P13#1 in the presence of SDS, LPSs or bacterial cells. (**A**,**B**) Emission spectra of aLuc-GKY20 and aLuc-P13#1, respectively, in sodium phosphate (NaP, blue line), SDS (red line) and LPSs (green line). (**C**,**D**) Emission spectra of aLuc-GKY20 and aLuc-P13#1, respectively, in NaP (blue line) or in the presence of *E. coli* (red line) *P. aeruginosa* (green line) and *S. aureus* (yellow line) cells. In each panel, the emissions in the presence of SDS, LPSs and bacterial cells were normalized to the emission in NaP.

This is typical of luciferin and is due to a well-known photoinduced deprotonation process (Figure S3 in Supplementary Materials). In the presence of SDS micelles, in addition to the major peak in the green region due to the phenolate, the emission spectra also show a broad peak in the blue region (430–450 nm). This emission, which is typical of the neutral form of luciferin (Figure S3 in Supplementary Materials), can only be observed in environments with very low water contents and no alternative proton acceptors, the sole conditions that can hinder the photoinduced deprotonation process [70], and confirms that the fluorophore is buried into the micelles. Furthermore, the blue shift of the phenolate emission (5–10 nm) confirms that the fluorophore is in a less polar environment. In the presence of the LPS micelles, the emission spectra show an even stronger blue shift of the phenolate emission (15–20 nm), but the blue emission is only visible in the case of Luc-P13#1, suggesting a deeper penetration into the micelles or a different orientation of the peptoid with respect to Luc-GKY20. In the presence of bacterial cells, we also found a large blue shift of the phenolate emission of 18–22 nm in the case of Luc-GKY20 and of about 15 nm in the case of Luc-P13#1. Once more, a small emission in the blue region was detected only in the case of Luc-P13#1, thus confirming the existence of small differences between the membrane-binding modes of Luc-GKY20 and Luc-P13#1. Very interestingly, the emission spectra were very similar in the presence of *E. coli*, *P. aeruginosa* and *S. aureus*, with only small variations in the emission wavelength (less than 5 nm). In the case of aLuc-GKY20 and aLuc-P13#1 (Figure 7), slightly larger blue shifts were detected upon binding to SDS, the LPSs and the bacterial cells (shifts of 15–19 nm, 25–28 nm and 19–21 nm, respectively), as is expected for the more solvatochromic aminoluciferin.

The binding of the P13#1 variants to SDS, the LPSs and the bacterial cells was also accompanied by strong positive or negative variations in the emission intensity. Usually, solvatochromic fluorophores show higher emission intensities in apolar environments, but luciferins are also sensitive to quenching, e.g., intramolecular, conformation-dependent quenching from tryptophan and tyrosine [70]. Therefore, the observed variations in the fluorescence emission are a net result deriving from these two opposite contributions. The reduction in the intensity of the emission was particularly evident in the presence of bacterial cells, likely because bacterial membranes contain proteins and other non-lipidic compounds (e.g., pigments) that might contribute to quenching (intermolecular quenching). We also studied the variations in the emission spectra of aLuc-GKY20 and aLuc-P13#1 in the presence of bacterial cells as functions of time (Figures S4 and S5 in Supplementary Materials). In all the cases, we found that the binding process was complete within 5 min. Therefore, the interactions of labeled GKY20 and P13#1 with bacterial cells are similar also from a kinetic point of view.

As LPSs are very strong proinflammatory molecules, CAMPs with high levels of affinity for these endotoxins show anti-inflammatory activities, acting as LPS scavengers. Therefore, we used Luc-P13#1 to determine the K_D_ values for submicellar (5 µg/mL) and micellar (40 µg/mL) LPSs from *E. coli* O111:B4 or *P. aeruginosa* 10. Fluorescent assays were carried out via incubating increasing concentrations of the labeled P13#1 and GKY20 with a constant concentration of LPSs. The samples were excited at 430 nm to selectively excite the phenolate form of luciferin, and the fluorescent emission was recorded in the 450–700 nm range. The total fluorescence was plotted as a function of the peptide/peptoid concentration, and the experimental data were fitted to a model equation. The fitting procedure provided the K_D_ values for the peptoid/LPS complexes and, in the case of the *E. coli* LPS (whose average MW is known), also provided an estimate of the binding stoichiometry (Table 3). The K_D_ values show that the labeled P13#1 and GKY20 bind LPSs from *E. coli* and *P. aeruginosa* with similar affinities. Interestingly, P13#1 and GKY20 bind submicellar LPSs with slightly higher affinities than micellar LPSs. This difference could be due to fact that submicellar LPSs are more accessible to peptides/peptoids than micellar LPSs. This explanation is supported by the binding stoichiometries: in the micellar state, 1–1.5 molecules of peptide/peptoid bind one LPS molecule, whereas in the case of a submicellar LPS, 4.4–5.2 molecules of peptide/peptoid bind one molecule of the LPS.

### 2.7. The Anti-Inflammatory Activity of P13#1 in Cell Cultures

**Figure 8:**
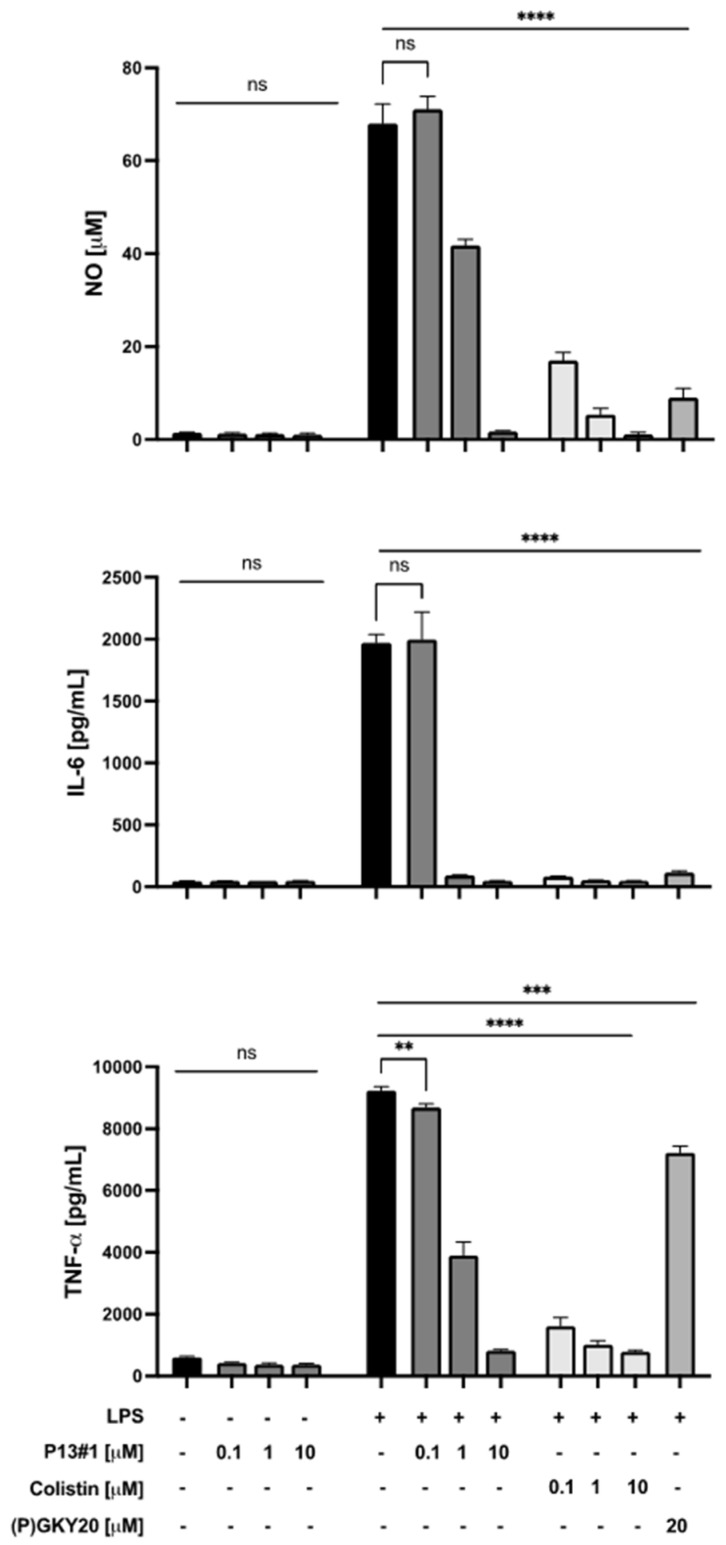
Effects of P13#1, (P)GKY20 and colistin on the release of NO and on the secretion of IL-6 and TNFα by Raw 264.7 murine macrophage cells stimulated with LPSs from *P. aerugionosa* 10. The experiments were performed in triplicate, and statistical analyses were carried out by using GraphPad Prism. The data presented are the mean values of three experiments ± S.Ds. The analysis was carried out using Student’s *t*-test (** *p* < 0.01, *** *p* < 0.001 and **** *p* < 0.0001) versus untreated cells and the LPS group.

Next, the LPS-scavenging activity of P13#1 was assayed in a cell culture model, i.e., Raw 264.7 murine macrophages. These cells, when stimulated with LPS, release several inflammation mediators including nitric oxide (NO) and cytokines [71]. Hence, Raw 264.7 cells were stimulated via exposure to LPS from *P. aeruginosa* 10 and treated with increasing concentrations (0.1, 1 and 10 µM) of P13#1 for 24 h. (P)GKY20 and colistin were used as positive controls. After the incubation, the nitric oxide (NO) released was measured via the Griess assay, and the pro-inflammatory cytokines interleukin-6 (IL-6) and tumor necrosis factor-α (TNFα) were quantified via an enzyme-linked immunosorbent assay (ELISA). It is worth noting that the biocompatibility of P13#1 was preliminarily tested on untreated Raw 264.7 cells by performing an MTT (3-(4,5-dimethylthiazol-2-yl)-2,5-diphenyltetrazolium bromide) assay, Griess assay and an ELISA. The collected results highlight that P13#1 does not exert a toxic effect against Raw 264.7 cells up to 10 µM (equivalent to 22 µg/mL of the chloride salt of P13#1) (Figure S6 in Supplementary Materials) and does not itself induce the release of proinflammatory factors (Figure 8). On the other hand, when administered to LPS-stimulated cells, P13#1 effectively inhibited the LPS-induced release of NO and cytokines (Figure 8). Very interestingly, at 10 µM, a non-toxic concentration, P13#1 completely re-established basal levels of pro-inflammatory mediators. Moreover, similar results were obtained on THP-1 human monocytes (Figure S7 in Supplementary Materials). Overall, the observed results indicate that P13#1 is a powerful LPS scavenger with an efficacy comparable to that of the benchmark peptide, colistin.

### 2.8. The In Vivo Efficacy of P13#1

**Figure 9:**
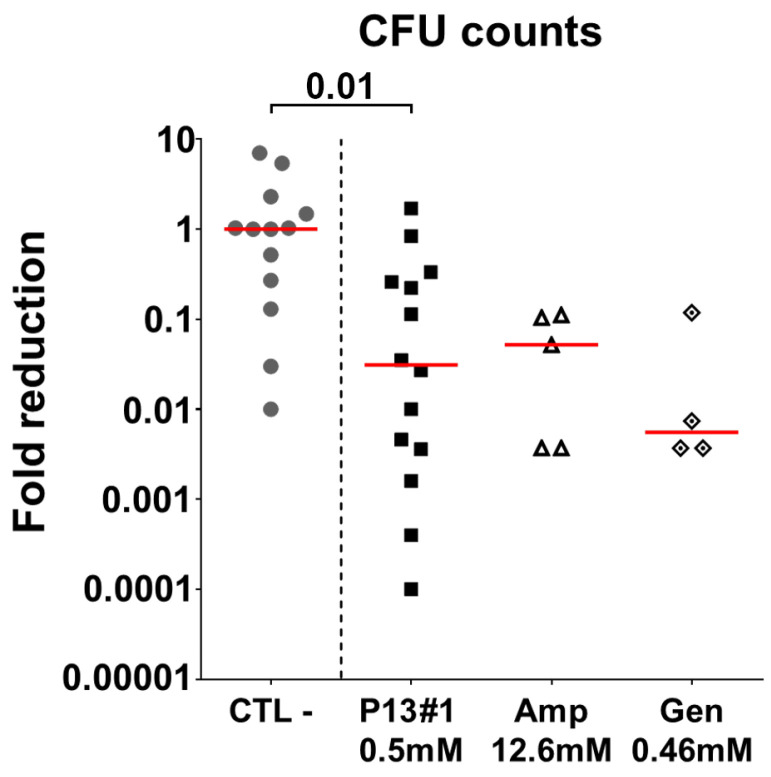
Fold reductions in the number of Colony Forming Units (CFUs) counted in air pouch washes with respect to the median value of the CFU counts of the negative control. Mice were inoculated with the *S. aureus* Newman strain injected into the pouch and then treated with PBS as a negative control (CTL-), P13 #1, ampicillin (Amp) or gentamycin (Gen), which were administered directly into the pouch. Each dot shows data from a single animal, the red line represents the median value and the data are from three independent experiments. The Mann–Whitney U-test was used to assess significance.

Encouraged by the results obtained in vitro, we sought to assess the antimicrobial efficacy of P13#1 in vivo in an air pouch model, a mouse model of subcutaneous infection in which bacteria are inoculated inside an air pouch obtained by injecting air into the back of a mouse [72]. The air pouch model allows one to mimic a localized infection that can then be treated via the topical administration of antimicrobial agents. As a model pathogen, we choose *S. aureus* as it is a common skin inhabitant and an opportunistic pathogen that frequently causes wound-related infections. In particular, *S. aureus* Newman was selected as it is a clinical strain isolated from a human infection [55] and its virulence in animal models is well known [73]. Mice were infected with this pathogen five days after the induction of the air pouch, and a single dose of the chosen antimicrobial, P13#1 (0.5 mM), ampicillin (12.6 mM) or gentamycin (0.46 mM), was injected into the air pouch 2 h after the infection. The mice were sacrificed after 48 h, and the bacterial load was determined via the CFU counts in washes of the air pouches. As shown in Figure 9, P13#1 protected the mice from *S. aureus* infection, reducing the CFU counts in the air pouch washes by almost 50-fold when compared to the negative control mice (pVal = 0.01).

**Figure 10:**
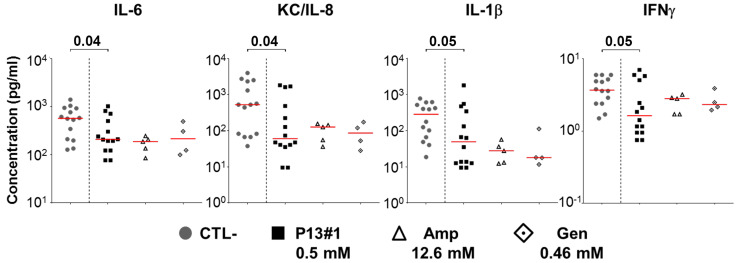
Quantification of cytokine concentrations in the pouches of animals infected with the *S. aureus* Newman strain and treated with PBS (CTL-), P13#1 or antibiotics. The cytokine concentration was measured at Luminex, and the data are expressed as pg/mL of wash. Each dot shows data from a single animal, the red line represents the median value and the data are from three independent experiments. The Mann–Whitney U-test was used to assess significance. Amp—ampicillin; Gen—gentamycin.

Interestingly, similar levels of protection were also observed with two well-known antibiotics, ampicillin, and gentamycin, underlying once more the potential of this compound as antimicrobial agent. The in vivo anti-inflammatory activity of P13#1 was then assessed by measuring the concentration of pro-inflammatory cytokines at the site of infection. As reported in Figure 10, the treatment with P13#1 contributed to significantly reducing the concentrations of several pro-inflammatory cytokines in the skin of the infected mice, and the results were comparable with those obtained when using the two antibiotics.

## 3. Materials and Methods

### 3.1. Materials and General Methods

The starting materials and reagents purchased from commercial suppliers were generally used without purification unless otherwise mentioned. *E. coli* BL21 (DE3) cells were purchased from Novagen (San Diego, CA, USA). The Ni SepharoseTM 6 Fast Flow was obtained from GE Healthcare (Uppsala, Sweden). All the other chemicals were obtained from Sigma-Aldrich (Milano, Italy). Reverse-phase high-performance liquid chromatography (RP-HPLC) analyses of P13#1 and CG-P13#1 were performed on a JASCO LC-NET II/ADC equipped with a JASCO Model PU-2089 Plus Pump and a JASCO MD-2010 Plus UV-vis multiple-wavelength detector set at 220 nm. The column used was a C18 reversed-phase analytical column (Waters, Bondapak, 10 μm, 125 Å, 3.9 mm × 300 mm), which was operated with linear gradients of acetonitrile (ACN) containing 0.1% trifluoroacetic acid (TFA) in H_2_O (0.1% TFA) over 30 min at a flow rate of 1.0 mL/min for the analytical runs. The high-resolution mass spectra (HRMS) were recorded on a Bruker Solarix XR Fourier-transform ion cyclotron resonance mass spectrometer (FTICR-MS) equipped with a 7T magnet, using matrix-assisted laser desorption/ionization (MALDI). Yields refer to chromatographically pure materials.

### 3.2. Solid-Phase Synthesis of P13#1

The Rink amide resin (4-(2’,4’-Dimethoxyphenyl-Fmoc-aminomethyl)-phenoxyacetamido-norleucyl-MBHA resin, copolymer styrene-1 % divinylbenzene (DVB), 100–200 mesh; 0.52 mmol g^−1^, 0.300 g, 0.156 mmol) was washed twice with 4.5 mL of CH_2_Cl_2_ and swelled in 4.5 mL of dry *N*,*N*-dimethylformamide (DMF) for 20 min (twice). The resin was Fmoc-deprotected using a 20% *v*/*v* piperidine solution in dry DMF (two treatments, 4.5 mL each for 20 min). The resin was then washed five times with 4.5 mL of DMF. The first sub-monomer was attached to the resin via the addition of bromoacetic acid (0.434 g, 3.12 mmol) in dry DMF (2.60 mL) and *N*,*N*’-diisopropylcarbodiimide (DIC, 580 μL, 3.74 mmol) on a shaker platform for 20 min at room temperature, followed by washing with DMF (3 × 1 min). A solution of *N*-Boc-1,4-butanediamine (1.00 M in dry DMF, 0.600 μL, 3.12 mmol) was added to the bromoacetylated resin. The mixture was left on a shaker platform for 20 min at room temperature; then, the resin was washed with DMF (3 × 1 min), followed by CH_2_Cl_2_ (3 × 1 min) and then again with DMF (3 × 1 min). Subsequent bromoacetylation reactions were accomplished by reacting the aminated oligomer with a solution of bromoacetic acid (0.434 g, 3.12 mmol) and DIC (580 μL, 3.74 mmol) in dry DMF (2.60 mL) for 20 min at room temperature. The filtrated resin was washed with DMF (3 × 1 min) and treated again with the proper amine under the same conditions reported above. This cycle of reactions was iterated until the target oligomer was obtained. The cleavage was performed by treating the resin with a solution of trifluoroacetic acid in m-cresol (95% *v*/*v*, 9.00 mL) on a shaker platform at room temperature for 2 h. The resin was then filtered away, and the filtrate was concentrated in vacuo to one-third of the initial volume. Then, the mixture was slowly added to 10.0 mL of stirred, cold diethyl ether. The white precipitate was centrifuged and washed several times with cold ether (10.0 mL). The linear oligomer (isolated as an amorphous white solid) was subjected to HRMS and HPLC (Figure S8 in Supplementary Materials).

P13#1: white amorphous solid, 0.291 g, 68%; tr 10.2 min. HRMS (MALDI-FT-ICR) *m*/*z*; [M + H]^+^ Calcd for C_107_H_155_N_20_O_14_^+^ 1945.2056; Found 1945.1884.

### 3.3. Solid-Phase Synthesis of CG-P13#1

Starting with on-bead P13#1, the oligomer was further elongated as follows. According to the monomeric protocol, a solution of Fmoc-Gly-OH (0.139 g, 0.468 mmol), hexafluorophosphate azabenzotriazole tetramethyl uronium (HATU) (0.172 g, 0.452 mmol) and *N*,*N*-diisopropylethylamine (DIPEA) (109 µL, 0.624 mmol) in dry DMF (1.06 mL) was added and left to shake for 90 min. The completion of the acylation reaction was verified by means of the Kaiser test. The monomer was subsequently deprotected using a 20% solution of piperidine in dry DMF (two treatments, 3 min and 7 min, respectively, 1.06 mL each) and then washed in the usual manner. Lastly, a solution of Fmoc-Cys(Trt)-OH (0.274 g, 0.468 mmol), HATU (0.172 g, 0.452 mmol) and DIPEA (109 µL, 0.624 mmol) in dry DMF (1.06 mL) was added and left to shake for 180 min. The completion of the acylation reaction was verified by means of the Kaiser test. The monomer was subsequently deprotected using a 20% solution of piperidine in dry DMF (two treatments, 3 min and 7 min, respectively, 1.06 mL each), and the resin was then washed as reported above. Cleavage was performed by treating the resin with a solution of trifluoroacetic acid in triethylsilane (95% *v*/*v*, 9.00 mL) on a shaker platform at room temperature for 2 h to obtain the simultaneous deprotection of sulfur. The resin was then filtered away, and the filtrate was concentrated in vacuo to one-third of the initial volume. The mixture was then slowly added to 10.0 mL of stirred, cold diethyl ether. The white precipitate was centrifuged and washed several times with cold ether (10.0 mL). The linear oligomer (isolated as an amorphous white solid) was subjected to HRMS and HPLC (Figure S9 in Supplementary Materials).

CG-P13#1: white amorphous solid, 0.0860 g, 19%; tr 12.2 min. HRMS (MALDI-TOF) *m*/*z*; [M + H]^+^ Calcd for C_112_H_163_N_22_O_16_S^+^ 2105.2363; Found 2105.3469.

### 3.4. Conformational Analysis of P13#1

The ZMM-MVM molecular modeling package (http://www.zmmsoft.ca, accessed on 30 August 2023) was used for the conformational analysis of P13#1. ZMM, a software that allows for conformational searches using generalized coordinates instead of conventional Cartesian coordinates [74] to make the conformational search faster, has already proved useful for the modeling of several biological macromolecules of different natures and sizes and in particular to model short peptides [75,76,77]. Atom–atom interactions were evaluated using the Amber force fields (cutoff distance = 8 Å). Electrostatic interactions were calculated using a distance-dependent dielectric constant with an initial value of 8.0 (ZMM parameters KEE = 2, EPS = 8.0). The energy calculations also included a water or octanol hydration component calculated using the methods developed by Karplus (ZMM parameter KES = 4) and Hopfinger and Battershell (ZMM parameter KES = 2), respectively, implemented in the ZMM software. Four starting conformations (left-handed and right-handed polyproline-helix-I-like and left-handed and right-handed polyproline-helix-II-like) were manually generated using PyMOL (https://pymol.org/2/, accessed on 30 August 2023). Each of the four structures was Monte-Carlo-energy-minimized (100,000 cycles per conformation) using both octanol and water solvations. To explore the stability of the conformations with mixed *trans* and *cis* peptide bonds, the HGRID function was used; 10,000 randomized structures were generated and energy-minimized (simple minimization; 1000 cycles for each random structure). The 2000 lowest-energy conformations were Monte-Carlo-minimized (1,200,000 total cycles).

### 3.5. The Purification of P13#1

P13#1 was purified via RP-HPLC, using a Jasco LC-4000 system equipped with PU-4086 semipreparative pumps and an MD-4010 photo diode array detector on a Europa Protein 300 C18 column (5 μm, 25 × 1) from Teknokroma (Barcelona, Spain), using 0.05% TFA in water (solvent A) and 0.05% TFA in ACN (solvent B) as solvents via linear gradient 1 (from 5% to 30% solvent B in 20 min, from 30% to 45% solvent B in 30 min, from 45% to 50% solvent B in 10 min and from 50% to 100% solvent B in 10 min, followed by isocratic elution at 100% solvent B for 10 min). The elution was monitored at 280 at a flow rate of 2 mL/min. The purified peptoid was lyophilized, dissolved in water and stored at −80 °C under a nitrogen atmosphere. The purity of P13#1 was evaluated via RP-HPLC (gradient 1, see above). Typically, the purity was >98%. The concentration of the purified P13#1 was determined via spectrophotometric analyses using an extinction coefficient ε_280_ = 1165 M^−1^ cm^−1^ (extinction coefficient of *O*-methyl-tyrosine [78], assumed to be identical to that of residue *N*mpe).

### 3.6. The Preparation of the Labeled Peptoids

CG-P13#1 (0.5 mg/mL final concentration) was labeled using 6-hydroxy-2-cyanobenzothiazole (CBT-OH) or 6-amino-2-cyanobenzothiazole (CBT-NH2) to obtain, respectively, Luc- and aLuc-P13#1. The labeling was performed following the procedure previously described in [70,79], with minor modifications. Briefly, reactions with cyanobenzothiazoles (CBTs) were performed in a 15 mM sodium phosphate buffer (NaP) at a pH of 7.4 which contained 2 M guanidine-HCl, 1 mM Tris(2-carboxyethyl)phosphine (TCEP) and 1 mM CBTs (10× stock solutions in DMF). The molar ratio of TCEP:CBT:free thiols was 4:4:1. The samples were incubated at 25 °C for 2 h in the dark under a nitrogen atmosphere. Control mixtures without peptoids were also prepared. The reaction yields were monitored via RP-HPLC on a C18 column, using linear gradient 1, as described above. The labeled peptoids were purified via C18 RP-HPLC (gradient 1). The purified, labeled peptoids were lyophilized and dissolved in water. The purity was verified as described for P13#1. Typically, the purity was >98%. Concentrations of the purified peptoids were determined via the Bradford assay, using an unlabeled peptoid as the standard.

### 3.7. The Production and Labelling of the Recombinant Peptides

Recombinant peptides (P)GKY20 and (C)GKY20 were prepared via the chemical hydrolysis of the reduced fusion proteins ONC-DCless-H6-(P)GKY20 and ONC-DCless-H6-(C)GKY20, respectively, expressed and purified as previously reported [57,79].

The labeling of (C)GKY20 with CBT-OH and CBT-NH2 was performed following the procedure previously described in [70,79]. The concentrations of the labeled peptides were determined via the Bradford assay, using unlabeled peptides as standards.

### 3.8. Antimicrobial Assay

The determination of the MIC values was performed using Gram(+) and Gram(−) bacteria via the broth microdilution method for antimicrobial peptides previously described in [40,57,80], with minor modifications. In detail, the assays were carried out in Nutrient Broth 0.5× (Difco, Detroit, Michigan), using sterile 96-well polypropylene microtiter plates (cat. 3879, Costar Corp., Cambridge, MA, USA). Bacterial strains were grown in Luria–Bertani (LB) medium overnight at 37 °C and then diluted in the Nutrient Broth at a final concentration of ~5 × 10^5^ CFU/mL per well. Twofold serial dilutions of P13#1 and (P)GKY20 were carried out in the test wells to obtain concentrations ranging from 50 μM to 0.05 μM. The plates were incubated overnight at 37 °C. The MIC value was taken as the lowest concentration that completely prevented visible growth [80]. Three independent experiments were performed for each MIC value. The antibiotic peptides polymyxin B and vancomycin (Merck KGaA, Darmstadt, Germany) were tested as controls (twofold serial dilutions, starting from a concentration of 64 µg/mL). The MIC values were measured on Gram(−) *Pseudomonas aeruginosa* PAO1, *Pseudomonas aeruginosa* KK27, *Pseudomonas aeruginosa* RP73, *Pseudomonas aeruginosa* AA2, *Pseudomonas aeruginosa* PA14, *Klebsiella pneumoniae* ATCC 700603, *Acinetobacter baumanii* ATCC 17878, *Burkholderia cenocepacea* LMG 18863, *Escherichia coli* ATCC 25922, *Salmonella typhimurium* ATCC 14028 and *Salmonella enteriditis* 706 RIVM) and the Gram(+) strains *Staphylococcus aureus* ATCC 6538P, *Staphylococcus aureus* WKZ2, *Staphylococcus aureus* Newman and *Enterococcus faecalis* ATCC 29212. To evaluate the bactericidal activity of P13#1 and (P)GKY20, the MBC was determined from the broth dilution of the MIC tests by subculturing cell mixtures on LB agar plates. The MBC was defined as the lowest concentration of an antibacterial agent that killed  ≥ 99.9% of bacterial cells. Synergy between P13#1/antibiotic combinations was assessed via the so-called checkerboard assay, a broth microdilution assay based on a two-dimensional array of serial dilutions of tested compounds. The experiments were carried out in 96-well plates on *P. aeruginosa* PAO1 (Gram-negative) and *S. aureus* ATCC 6538P (Gram-positive). P13#1 was tested in combination with colistin, tobramycin, ciprofloxacin and meropenem. The plates were incubated for 16 h at 37 °C. The fractional inhibitory concentration index (FIC_i_) was calculated as follows: FIC_A_ + FIC_B_, where FIC_A_ = MIC of drug A in combination/MIC of drug A alone, and FIC_B_ = MIC of drug B in combination/MIC of drug B alone.

### 3.9. Anti-Biofilm Assay

The anti-biofilm activity of P13#1 and LL-37 was tested on the *P. aeruginosa* strains PAO1 and RP73. The bacteria were grown overnight in Luria–Bertani (LB) medium at 37 °C. The overnight cultures were diluted to 1 × 10^8^ CFU/mL and incubated in BM2 biofilm-adjusted medium [81] (62 mM potassium phosphate buffer pH 7.0, 7 mM (NH_4_)_2_SO_4_, 2 mM MgSO4, 10 μM FeSO4, 0.4% (*w*/*v*) glucose) in polystyrene 96-well microtiter plates (Corning^®^, New York, NY, USA) for 24 h at 37 °C. The one-day-old biofilms were than treated with increasing concentrations of P13#1 and LL-37 (0.39–30 µM) to evaluate their ability to eradicate a pre-formed biofilm after 4 h of incubation time at 37 °C. At the end of the incubation, planktonic cultures were removed from the wells. After washing the cultures three times with sterile PBS, each biofilm mass was stained using 0.1% crystal violet for 20 min. The excess crystal violet was eliminated via three successive washes with sterile water. Finally, the crystal violet was solubilized using 30% acetic acid, and the optical absorbance values were determined at 595 nm using a microtiter plate reader (Synergy™ H4, BioTek, Winooski, VT, USA). The metabolic activity of the cells after treatment was evaluated via a Cell Proliferation Kit II assay (XTT) (Merck KGaA, Darmstadt, Germany). The planktonic cells were removed, and the plates were thoroughly washed three times with PBS. The assay was carried out in 100 μL of PBS supplemented with 50 μL of XTT solution. The plates were incubated in the dark for 3–6 h at 37 °C. The reduction of the tetrazolium salt into the orange formazan dye by the metabolically active cells was measured at 490 nm using a microtiter plate reader (SynergyTM H4, BioTek, USA). The viability was compared to controls carried out in the absence of an antimicrobial agent. MBEC values were determined by counting the viable bacterial cells inside the biofilm’s structure upon disrupting the biofilm with 1% Triton X-100 for 10 min. The cells were serially diluted in the LB medium and plated on LB/agar plates. After incubating for 16 h at 37 °C, the CFUs were counted. The percentages of surviving cells were calculated as follow: (CFU treated sample/CFU untreated sample) × 100.

### 3.10. The Interaction of Labeled Species with LPSs and SDS

The binding of the labeled P13#1 and GKY20 (2 μM) to LPSs (200 μg/mL) from *E. coli* 0111:B4 and SDS (25 mM) was performed in a 10 mM NaP buffer at a pH of 7.4 at 25 °C. The mixtures were equilibrated at 25 °C for 30 min before recording the emission and excitation spectra. Fluorescent analyses were carried out in 96-well polystyrene microtiter plates containing 100 μL of the mixtures. The spectra were recorded using a SynergyTM H4 microplate reader (BioTek Instruments Inc., Winooski, VT, USA). The excitation wavelengths were set to 330 nm (Luc-P13#1 and Luc-GKY20, phenol form), 430 nm (Luc-P13#1 and Luc-GKY20, phenolate form) and 363 nm (aLuc-P13#1 and aLuc-GKY20). The excitation spectra were recorded between 200 nm and 500 nm (em. = 539 nm for Luc-P13#1 and Luc-GKY20; em. = 525 nm for aLuc-P13#1 and aLuc-GKY20).

### 3.11. The Interaction of the Labeled Species with Bacterial Cells

Bacterial strains (*E. coli* ATCC 25922, *P. aeruginosa* PAO1 and *S. aureus* ATCC 6538P) were cultured in LB medium at 37 °C overnight. The cultures were diluted 1:100 in fresh LB medium, and the bacteria were grown until an optical density of 1 OD600 was reached. The cells were collected via centrifugation at 8000× *g* for 5 min at 4 °C, washed three times in a 10 mM NaP buffer at a pH of 7.4 and suspended at a concentration of 1 OD600 (10× cell stock solution) in the same buffer. The bacteria mixtures were stored on ice until use. The binding of labeled P13#1 and GKY20 to the cells was performed in 100 µL of the 10 mM NaP buffer at a pH of 7.4 in the presence of 0.1 OD600 bacterial cells and 2 μM of labeled P13#1 or GKY20. The reactions were initiated by adding the cells, and the emission spectra were recorded at 25 °C at 0–5–10–15–20 min (Luc-labeled species, ex. = 330 and 430 nm; aLuc-labeled species, ex. = 363 nm).

### 3.12. K_D_ Calues and Binding Stoichiometries toward LPS

The binding of Luc-P13#1 and Luc-GKY20 to LPSs was assayed using LPSs from *E. coli* 0111:B4 and *P. aeruginosa* 10. The K_D_ values of Luc-P13#1 and Luc-GKY20 for the LPSs from *E. coli* 0111:B4 were determined at micellar (40 μg/mL, corresponding to about 4 μM, considering a MW = 10,000 [82]) and sub-micellar (5 μg/mL ≈ 0.5 μM) LPS concentrations in the presence of variable concentrations of Luc-P13#1 and Luc-GKY20 which ranged from 0.25 to 18 μM and from 0.01 to 5.5 μM, respectively. The LPSs from *P. aeruginosa* 10 were used only at 5 μg/mL. The assays were carried out in 96-well polystyrene microtiter plates containing 100 μL of binding mixtures in a 10 mM NaP buffer at a pH of 7.4. The spectra were recorded using a SynergyTM H4 microplate reader. The mixtures were incubated for 30 min before the emission spectra were recorded via excitation at 430 nm (phenolate form). The variation in the total fluorescence (480–650 nm) was reported as a function of the peptoid/peptide concentration, and the data were fitted to the model using Graphpad Prism, as described [70].

### 3.13. MTT Assay

The cytotoxic effects of P13#1 on Raw 264.7 cells were determined by performing an MTT assay designed to be used for the spectrophotometric quantification of cell proliferation. Briefly, 2 × 10^4^ cells were seeded into a 96-well plate and incubated at 37 °C in the presence of 5% CO_2_. The medium was then replaced with 100 μL of a fresh medium containing the peptide solution at a final concentration ranging from 0.15 to 40 μM/well. After 6, 24 and 48 h of incubation at 37 °C, the media were removed, and 100 μL of tetrazolium MTT diluted at 0.5 mg/mL in Dulbecco’s modified Eagle’s medium (DMEM) purchased from Lonza (Basel, Switzerland) without red phenol was added. After 4 h of incubation at 37 °C, the resulting insoluble formazan salts were solubilized in 0.04 M HCl in anhydrous isopropanol and quantified by measuring the absorbance at λ = 570 nm, using an automatic plate reader spectrophotometer (SynergyTM H4, BioTek, USA). Cell survival was expressed as the mean of the percentage value compared to the control. Analyses were performed at least three times.

### 3.14. The Immune-Modulatory Activity of P13#1

The ability of P13#1 to modulate cytokines and the production of nitric oxide in RAW 264.7 cells was measured via an ELISA (enzyme-linked immunosorbent assay) and a Griess assay, respectively. Cells (2 × 10^4^ cells/well) were seeded into 96-well microtiter plates. The next day, the culture medium was discarded and replaced with a fresh medium containing either (i) a mixture of P13#1 (0.1, 1 10 μM) and LPSs from *P. aeruginosa* 10 (co-incubation), (ii) only P13 #1 (0.1, 1, 10 μM) or (iii) only LPSs from *P. aeruginosa* 10. The inhibition of LPSs exerted by P13 #1 has been compared to 20 μM of (P)GKY20 and 0.1–1–10 μM of colistin. Cell supernatants were collected after 24 h of incubation at 37 °C and 5% CO_2_. The release of TNFα and the release of IL-6 were measured using DuoSet ELISA kits (R & D Systems), following the protocols provided by the manufacturer. All the samples were centrifuged briefly at 5000 rpm for 3 min at room temperature to remove cell debris prior to their use. The microtiter plates were read at 450 nm, using 550 nm as a reference wavelength to correct for the optical imperfections of the microtiter plate. The nitrite concentrations were determined via a colorimetric reaction using the Griess Reagent Kit for nitrite quantitation (Invitrogen™). Briefly, cell culture supernatants were mixed with equal volumes of *N*-(1-naphthyl)ethylenediamine (Component A) and sulfanilic acid (Component B) to form the Griess Reagent and incubated for 30 min at room temperature. The absorbance was measured at 548 nm using a 96-well microplate reader (SynergyTM H4, BioTek, USA).

### 3.15. In Vivo Assays

For the entire experimental period, the animals were kept at an AAALAC-accredited facility. Upon their arrival, animals were randomly distributed into different experimental groups in individually ventilated cages (IVC, Sealsafe Plus GM500 by Tecniplast). The acclimation period lasted for 5 days. At the end of the acclimation period, each animal was identified via an individual tattoo. All the animals had ad libitum access to GMP-grade food (Mucedola 4RF25 TOP CERTIFICATE) and bottled, filtered tap water. Certified, irradiated cellulose bags containing Mucedola SCOBIS UNO bedding and carboard tunnels (ANTRUM) or plexiglass mouse houses were provided within the cages. A few food pellets and wood blocks in the cages were also used as enrichment for foraging and additional gnawing. Cage and bedding changes were performed once every two weeks in agreement with the cage supplier’s indications and the AAALAC indications. The air supplied via the IVC was 100% fresh air filtered by an EPA filter in the IVC system, with 60–75 air changes per hour. The animal room conditions were as follows: a temperature of 21 °C (+/−3 °C), a relative humidity of 50% (range 30–70%) and a 12 h/12 h light/dark cycle. The pressure, temperature and relative humidity were recorded continuously using room probes, while the IVC system recorded the performance of the individual motors. The light-cycle setting was ensured via a qualified validated, alarmed system.

The experiments were carried out as previously reported [72]. Briefly, two 3 mL subcutaneous, dorsolateral ml injections of air (on days 0 and 3) were performed to generate a single pouch into which 10^7^ CFU (a volume of 500 µL) of the *S. aureus* Newman strain were inoculated on day 5. Two hours after infection, the mice were treated directly in the air pouch with the reported concentrations of the antimicrobial compounds. The mice were sacrificed 48 h after the challenge, and the pouches were injected with 1 mL of sterile PBS which was immediately withdrawn using the same syringe to perform CFU counting and cytokine titrations.

## 4. Conclusions

CAMPs are widely recognized as some of the most attractive solutions to the antibiotic resistance problem, in fact, their peculiar mechanism of action makes a rapid insurgence of resistant strains more unlikely [3,4,5,6,7]. Until now, however, it has been difficult to translate CAMPs into clinical practice due to some relevant drawbacks; first of all, their sensitivity to proteases results in short in vivo half-lives and limited administration routes. Furthermore, the high costs of producing CAMPs would prevent industrial scale-up.

We designed the peptoid P13#1 to attempt to overcome these two issues. The peculiar structures of peptoids make them intrinsically resistant to proteases, and the sub-monomer method makes their production easier and cheaper production. Moreover, we chose to use only residues without stereogenic centers and groups that might have made the synthesis more complex, increased production costs and reduced yields.

We designed P13#1 to mimic the general structural and functional features of CAMPs and of vertebrate cathelicidins without aiming to reproduce a specific CAMP. Nonetheless, it shows similarities with some of the shortest natural cathelicidins, as discussed in Section 2.1. As anticipated, P13#1 shows biological activities similar or even better than those of the reference CAMPs. It shows bactericidal and wide-spectrum antimicrobial activities similar to cathelicidins and cryptic cathelicidin-like peptides, a greater antibiofilm activity than that of LL-37 and an ability to neutralize the proinflammatory activities of LPSs which is comparable to the activity of colistin. The characterization of the labeled versions has also shown that at the molecular level, P13#1 has a behavior which is very similar to that of a cryptic cathelicidin-like CAMP. In an air pouch model of subcutaneous infection with *S. aureus*, P13#1 showed antimicrobial and anti-inflammatory activities comparable to those of ampicillin and gentamicin. Although additional experiments must be performed to evaluate its in vivo efficacy in different models of infection and routes of administration, P13#1 can be considered a potential antimicrobial peptidomimetic candidate for therapeutic applications.

## Figures and Tables

**Table 1:** Antimicrobial activity.

	MIC (MBC) ^1^		MIC	
	(μM)		(μM)	
Bacterial Strain	P13#1	(P)GKY20	Vanc ^2^	Pol B ^2^
**Gram(−)**				
*Pseudomonas aeruginosa* PAO1	1.56 (1.56)	3.12 (3.12)		0.18
*Pseudomonas aeruginosa* KK27	1.56 (1.56)	nd ^3^		0.36
*Pseudomonas aeruginosa* RP73	1.56 (1.56)	1.56 (1.56)		0.09
*Pseudomonas aeruginosa* AA2	1.56 (1.56)	nd		0.36
*Pseudomonas aeruginosa* PA14	1.56 (1.56)	6.25 (6.25)		0.18
*Klebsiella pneumoniae* ATCC 700603	1.56 (1.56)	6.25 (6.25)		0.36
*Acinetobacter baumanii* ATCC 17878	1.56 (1.56)	3.12 (3.12)		0.18
*Burkholderia cenocepacea* LMG 18863	>50	>50		>46.2
*Escherichia coli* ATCC 25922	1.56 (1.56)	6.25 (6.25)		0.72
*Salmonella typhimurium* ATCC 14028	1.56 (1.56)	3.12 (3.12)		0.36
*Salmonella enteriditis* 706 RIVM	1.56 (1.56)	6.25 (6.25)		0.72
**Gram(+)**				
*Staphylococcus aureus* ATCC 6538P	0.78 (0.78)	1.56 (1.56)	0.34	
*Staphylococcus aureus* WKZ2 (MRSA)	1.56 (1.56)	nd	0.34	
*Staphylococcus aureus* Newman	3.12 (3.12)	6.25 (6.25)	0.17	
*Enterococcus faecalis* ATCC 29212	1.56 (1.56)	1.56 (1.56)	0.34	

^1^ Data were obtained from a minimum of three independent experiments. ^2^ Vanc—vancomycin; Pol B—polymyxin B. ^3^ nd: not determined.

**Table 2:** Combination therapy analysis.

Bacterial Strains	Antibiotic	MIC (μg/mL)	ΣFIC_i_ ^1^
			P13#1
**Gram(−)**			
*Pseudomonas aeruginosa* PAO1	**Tobramycin**	0.0078	0.626
	**Ciprofloxacin**	0.125	0.750
	**Colistin**	0.25	0.376
	**Meropenem**	0.25	2
**Gram(+)**			
*Staphylococcus aureus* ATCC 6538P	**Tobramycin**	0.00097	0.750
	**Ciprofloxacin**	0.125	1
	**Meropenem**	0.0625	2

^1^ Fractional inhibitory concentration index (FIC_i_) values were determined for P13#1 in combination with antibiotics on Gram(+) and Gram(−) strains. The indexes were obtained from a minimum of three independent experiments, each one carried out in triplicate.

**Table 3:** K_D_ values and binding stoichiometries.

Peptide/ Peptoid	LPS*_Ec_* ^1^ (µg/mL)	K_D_ (µM)	[Binding Sites] (µM)	Stoichiometry ^2^ (Peptoid:LPS)
**Luc-P13#1**	40	0.121 (±0.032)	4.53 (±0.123)	1.13:1
**Luc-P13#1**	5	0.085 (±0.021)	2.21 (±0.107)	4.42:1
**Luc-GKY20**	40	0.382 (±0.030)	6.12 (±0.109)	1.53:1
**Luc-GKY20**	5	0.109 (±0.022)	2.62 (±0.153)	5.24:1
	**LPS*_Pa_* ^3^** **(µg/mL)**			
**Luc-P13#1**	5	0.032 (±0.0085)	1.91 (±0.049)	-
**Luc-GKY20**	5	0.054 (±0.013)	2.1 (±0.08)	-

^1^ LPS*_Ec_*: LPS from *Escherichia coli* 0111:B4. ^2^ Stoichiometry was determined assuming an average MW = 10,000 Da for the *E. coli* LPS. ^3^ LPS*_Pa_*: LPS from *Pseudomonas aeruginosa* 10.

## Data Availability

The data presented in this study are available upon request from the corresponding author. The authors confirm that all relevant data are included in the article.

## Supplementary Materials

The following supporting information can be downloaded at: https://www.mdpi.com/article/10.3390/ph16101386/s1, Figure S1: Representative Monte-Carlo-minimized structures of P13#1 using water solvation; Figure S2: Comparison between representative Monte-Carlo-minimized structures of P13#1 using octanol solvation and the NMR structures of bovine indolicidin; Figure S3: Structure and fluorescence properties of the labeled peptoids and peptides; Figure S4: Time variations in the emission spectra after excitation at 330 nm of Luc-GKY20 and Luc-P13#1 in the presence of bacterial cells; Figure S5: Time variations in the emission spectra of aLuc-GKY20 and aLuc-P13#1 in the presence of bacterial cells; Figure S6: Viability of Raw 264.7 murine macrophage cells treated with P13#1; Figure S7: Effects of (P)GKY20 and P13#1 on the release of TNFα from human THP-1 cells treated with LPS from *P. aeruginosa* 10; Figure S8: HPLC analysis of P13#1; Figure S9: HPLC analysis of CG-P13#1.

## References

[1] Wiesner J., Vilcinskas A. (2010). Antimicrobial peptides: The ancient arm of the human immune system. Virulence.

[2] Mansour S.C., Pena O.M., Hancock R.E.W. (2014). Host defense peptides: Front-line immunomodulators. Trends Immunol..

[3] De Breij A., Riool M., Cordfunke R.A., Malanovic N., De Boer L., Koning R.I., Ravensbergen E., Franken M., Van Der Heijde T., Boekema B.K. (2018). The antimicrobial peptide SAAP-148 combats drug-resistant bacteria and biofilms. Sci. Transl. Med..

[4] Narayana J.L., Huang H.N., Wu C.J., Chen J.Y. (2015). Efficacy of the antimicrobial peptide TP4 against *Helicobacter pylori* infection: In vitro membrane perturbation via micellization and in vivo suppression of host immune responses in a mouse model. Oncotarget.

[5] Barreto-Santamaría A., Rivera Z.J., García J.E., Curtidor H., Patarroyo M.E., Patarroyo M.A., Arévalo-Pinzón G. (2020). Shorter antibacterial peptide having high selectivity for *E. coli* membranes and low potential for inducing resistance. Microorganisms.

[6] Chatupheeraphat C., Peamchai J., Luk-in S., Eiamphungporn W. (2023). Synergistic effect and antibiofilm activity of the antimicrobial peptide K11 with conventional antibiotics against multidrug-resistant and extensively drug-resistant *Klebsiella pneumoniae*. Front. Cell. Infect. Microbiol..

[7] Chou S., Wang J., Shang L., Akhtar M.U., Wang Z., Shi B., Feng X., Shan A. (2019). Short, symmetric-helical peptides have narrow-spectrum activity with low resistance potential and high selectivity. Biomater. Sci..

[8] Pamp S.J., Gjermansen M., Johansen H.K., Tolker-Nielsen T. (2008). Tolerance to the antimicrobial peptide colistin in *Pseudomonas aeruginosa* biofilms is linked to metabolically active cells, and depends on the pmr and mexAB-oprM genes. Mol. Microbiol..

[9] Chung P.Y., Khanum R. (2017). Antimicrobial peptides as potential anti-biofilm agents against multidrug-resistant bacteria. J. Microbiol. Immunol. Infect..

[10] de la Fuente-Núñez C., Reffuveille F., Haney E.F., Straus S.K., Hancock R.E.W. (2014). Broad-Spectrum Anti-biofilm Peptide That Targets a Cellular Stress Response. PLoS Pathog..

[11] Jacobsen A.S., Jenssen H. (2012). Human cathelicidin LL-37 prevents bacterial biofilm formation. Future Med. Chem..

[12] Martin L., van Meegern A., Doemming S., Schuerholz T. (2015). Antimicrobial peptides in human sepsis. Front. Immunol..

[13] Di Grazia A., Cappiello F., Cohen H., Casciaro B., Luca V., Pini A., Di Y.P., Shai Y., Mangoni M.L. (2015). D-Amino acids incorporation in the frog skin-derived peptide esculentin-1a(1-21)NH_2_ is beneficial for its multiple functions. Amino Acids.

[14] Quercini L., Brunetti J., Riolo G., Bindi S., Scali S., Lampronti I., D’Aversa E., Wronski S., Pollini S., Gentile M. (2020). An antimicrobial molecule mitigates signs of sepsis in vivo and eradicates infections from lung tissue. FASEB J..

[15] Cresti L., Cappello G., Vailati S., Melloni E., Brunetti J., Falciani C., Bracci L., Pini A. (2023). In Vivo Efficacy and Toxicity of an Antimicrobial Peptide in a Model of Endotoxin-Induced Pulmonary Inflammation. Int. J. Mol. Sci..

[16] Brunetti J., Carnicelli V., Ponzi A., Di Giulio A., Lizzi A.R., Cristiano L., Cresti L., Cappello G., Pollini S., Mosconi L. (2020). Antibacterial and anti-inflammatory activity of an antimicrobial peptide synthesized with D amino acids. Antibiotics.

[17] Mora P., Mas-Moruno C., Tamboredo S., Cruz L.J., Pérez-Payá E., Albericio F. (2006). Design of a minimized cyclic tetrapeptide that neutralizes bacterial endotoxins. J. Pept. Sci..

[18] van Os N., Javed A., Broere F., van Dijk A., Balhuizen M.D., van Eijk M., Rooijakkers S.H.M., Bardoel B.W., Heesterbeek D.A.C., Haagsman H.P. (2022). Novel insights in antimicrobial and immunomodulatory mechanisms of action of PepBiotics CR-163 and CR-172. J. Glob. Antimicrob. Resist..

[19] Javed A., Slingerland C.J., Wood T.M., Martin N.I., Broere F., Weingarth M.H., Veldhuizen E.J.A. (2023). Chimeric Peptidomimetic Antibiotic Efficiently Neutralizes Lipopolysaccharides (LPS) and Bacteria-Induced Activation of RAW Macrophages. ACS Infect. Dis..

[20] van Harten R.M., Veldhuizen E.J.A., Haagsman H.P., Scheenstra M.R. (2022). The cathelicidin CATH-2 efficiently neutralizes LPS- and *E. coli*-induced activation of porcine bone marrow derived macrophages. Vet. Immunol. Immunopathol..

[21] Ibrahim H.R., Hamasaki K., Miyata T. (2017). Novel peptide motifs from lysozyme suppress pro-inflammatory cytokines in macrophages by antagonizing toll-like receptor and LPS-scavenging action. Eur. J. Pharm. Sci..

[22] Tran T.B., Velkov T., Nation R.L., Forrest A., Tsuji B.T., Bergen P.J., Li J. (2016). Pharmacokinetics/pharmacodynamics of colistin and polymyxin B: Are we there yet?. Int. J. Antimicrob. Agents.

[23] Molchanova N., Hansen P.R., Franzyk H. (2017). Advances in development of antimicrobial peptidomimetics as potential drugs. Molecules.

[24] Dohm M.T., Kapoor R., Barron A.E. (2012). Peptoids: Bio-Inspired Polymers as Potential Pharmaceuticals. Curr. Pharm. Des..

[25] Miller S.M., Simon R.J., Ng S., Zuckermann R.N., Kerr J.M., Moos W.H. (1994). Proteolytic studies of homologous peptide and N-substituted glycine peptoid oligomers. Bioorg. Med. Chem. Lett..

[26] Weiser L.J., Santiso E.E. (2017). Molecular modeling studies of peptoid polymers. AIMS Mater. Sci..

[27] Chongsiriwatana N.P., Patch J.A., Czyzewski A.M., Dohm M.T., Ivankin A., Gidalevitz D., Zuckermann R.N., Barron A.E. (2008). Peptoids that mimic the structure, function, and mechanism of helical antimicrobial peptides. Proc. Natl. Acad. Sci. USA.

[28] Lee J., Kang D., Choi J., Huang W., Wadman M., Barron A.E., Seo J. (2018). Effect of side chain hydrophobicity and cationic charge on antimicrobial activity and cytotoxicity of helical peptoids. Bioorg. Med. Chem. Lett..

[29] Patch J.A., Barron A.E. (2003). Helical peptoid mimics of magainin-2 amide. J. Am. Chem. Soc..

[30] Bolt H.L., Eggimann G.A., Jahoda C.A.B., Zuckermann R.N., Sharples G.J., Cobb S.L. (2017). Exploring the links between peptoid antibacterial activity and toxicity. Medchemcomm.

[31] De Riccardis F. (2020). The Challenge of Conformational Isomerism in Cyclic Peptoids. Eur. J. Org. Chem..

[32] Nam H.Y., Choi J., Kumar S.D., Nielsen J.E., Kyeong M., Wang S., Kang D., Lee Y., Lee J., Yoon M.H. (2020). Helicity Modulation Improves the Selectivity of Antimicrobial Peptoids. ACS Infect. Dis..

[33] Shagaghi N., Palombo E.A., Clayton A.H.A., Bhave M. (2016). Archetypal tryptophan-rich antimicrobial peptides: Properties and applications. World J. Microbiol. Biotechnol..

[34] Loffredo M.R., Ghosh A., Harmouche N., Casciaro B., Luca V., Bortolotti A., Cappiello F., Stella L., Bhunia A., Bechinger B. (2017). Membrane perturbing activities and structural properties of the frog-skin derived peptide Esculentin-1a(1-21)NH_2_ and its Diastereomer Esc(1-21)-1c: Correlation with their antipseudomonal and cytotoxic activity. Biochim. Biophys. Acta-Biomembr..

[35] Jia F., Wang J., Peng J., Zhao P., Kong Z., Wang K., Yan W., Wang R. (2017). D-amino acid substitution enhances the stability of antimicrobial peptide polybia-CP. Acta Biochim. Biophys. Sin..

[36] Shai Y., Oren Z. (1996). Diastereomers of cytolysins, a novel class of potent antibacterial peptides. J. Biol. Chem..

[37] Bosi S., Fabbro A., Ballerini L., Prato M. (2013). Carbon nanotubes: A promise for nerve tissue engineering?. Nanotechnol. Rev..

[38] Dürr U.H.N., Sudheendra U.S., Ramamoorthy A. (2006). LL-37, the only human member of the cathelicidin family of antimicrobial peptides. Biochim. Biophys. Acta-Biomembr..

[39] Pane K., Durante L., Crescenzi O., Cafaro V., Pizzo E., Varcamonti M., Zanfardino A., Izzo V., Di Donato A., Notomista E. (2017). Antimicrobial potency of cationic antimicrobial peptides can be predicted from their amino acid composition: Application to the detection of “cryptic” antimicrobial peptides. J. Theor. Biol..

[40] Pane K., Sgambati V., Zanfardino A., Smaldone G., Cafaro V., Angrisano T., Pedone E., Di Gaetano S., Capasso D., Haney E.F. (2016). A new cryptic cationic antimicrobial peptide from human apolipoprotein E with antibacterial activity and immunomodulatory effects on human cells. FEBS J..

[41] Bosso A., Pirone L., Gaglione R., Pane K., Del Gatto A., Zaccaro L., Di Gaetano S., Diana D., Fattorusso R., Pedone E. (2017). A new cryptic host defense peptide identified in human 11-hydroxysteroid dehydrogenase-1 β-like: From in silico identification to experimental evidence. Biochim. Biophys. Acta-Gen. Subj..

[42] Pane K., Cafaro V., Avitabile A., Torres M.D.T., Vollaro A., De Gregorio E., Catania M.R., Di Maro A., Bosso A., Gallo G. (2018). Identification of Novel Cryptic Multifunctional Antimicrobial Peptides from the Human Stomach Enabled by a Computational-Experimental Platform. ACS Synth. Biol..

[43] Gaglione R., Dell’Olmo E., Bosso A., Chino M., Pane K., Ascione F., Itri F., Caserta S., Amoresano A., Lombardi A. (2017). Novel human bioactive peptides identified in Apolipoprotein B: Evaluation of their therapeutic potential. Biochem. Pharmacol..

[44] Torres M.D.T., Melo M.C.R., Crescenzi O., Notomista E., de la Fuente-Nunez C. (2022). Mining for encrypted peptide antibiotics in the human proteome. Nat. Biomed. Eng..

[45] Lone A., Arnous A., Hansen P.R., Mojsoska B., Jenssen H. (2020). Synthesis of Peptoids Containing Multiple Nhtrp and Ntrp Residues: A Comparative Study of Resin, Cleavage Conditions and Submonomer Protection. Front. Chem..

[46] Rozek A., Friedrich C.L., Hancock R.E.W. (2000). Structure of the Bovine Antimicrobial Peptide Indolicidin Bound to Dodecylphosphocholine and Sodium Dodecyl Sulfate Micelles. Biochemistry.

[47] Maayan G., Ward M.D., Kirshenbaum K. (2009). Folded biomimetic oligomers for enantioselective catalysis. Proc. Natl. Acad. Sci. USA.

[48] Huang M.L., Shin S.B.Y., Benson M.A., Torres V.J., Kirshenbaum K. (2012). A comparison of linear and cyclic peptoid oligomers as potent antimicrobial agents. ChemMedChem.

[49] Mikkelsen H., McMullan R., Filloux A. (2011). The *Pseudomonas aeruginosa* reference strain PA14 displays increased virulence due to a mutation in ladS. PLoS ONE.

[50] Bragonzi A., Paroni M., Nonis A., Cramer N., Montanari S., Rejman J., Di Serio C., Döring G., Tümmler B. (2009). *Pseudomonas aeruginosa* microevolution during cystic fibrosis lung infection establishes clones with adapted virulence. Am. J. Respir. Crit. Care Med..

[51] Bragonzi A., Wiehlmann L., Klockgether J., Cramer N., Worlitzsch D., Döning G., Tümmler B. (2006). Sequence diversity of the mucABD locus in *Pseudomonas aeruginosa* isolates from patients with cystic fibrosis. Microbiology.

[52] Di Lorenzo F., Silipo A., Bianconi I., Lore’ N.I., Scamporrino A., Sturiale L., Garozzo D., Lanzetta R., Parrilli M., Bragonzi A. (2015). Persistent cystic fibrosis isolate *Pseudomonas aeruginosa* strain RP73 exhibits an under-acylated LPS structure responsible of its low inflammatory activity. Mol. Immunol..

[53] Bianconi I., Jeukens J., Freschi L., Alcalá-Franco B., Facchini M., Boyle B., Molinaro A., Kukavica-Ibrulj I., Tümmler B., Levesque R.C. (2015). Comparative genomics and biological characterization of sequential *Pseudomonas aeruginosa* isolates from persistent airways infection. BMC Genom..

[54] Bloemendaal A.L.A., Brouwer E.C., Fluit A.C. (2010). Methicillin resistance transfer from *Staphylocccus epidermidis* to methicillin-susceptible *Staphylococcus aureus* in a patient during antibiotic therapy. PLoS ONE.

[55] Lorenz L.L., Duthie E.S. (1952). Staphylococcal Coagulase: Mode of Action and Antigenicity. J. Gen. Microbiol..

[56] Kasetty G., Papareddy P., Kalle M., Rydengård V., Mörgelin M., Albiger B., Malmsten M., Schmidtchen A. (2011). Structure-activity studies and therapeutic potential of host defense peptides of human thrombin. Antimicrob. Agents Chemother..

[57] Pane K., Durante L., Pizzo E., Varcamonti M., Zanfardino A., Sgambati V., Di Maro A., Carpentieri A., Izzo V., Di Donato A. (2016). Rational design of a carrier protein for the production of recombinant toxic peptides in *Escherichia coli*. PLoS ONE.

[58] Loutet S.A., Valvano M.A. (2011). Extreme antimicrobial peptide and polymyxin B resistance in the genus Burkholderia. Front. Cell. Infect. Microbiol..

[59] Ghimire J., Guha S., Nelson B.J., Morici L.A., Wimley W.C. (2022). The Remarkable Innate Resistance of *Burkholderia bacteria* to Cationic Antimicrobial Peptides: Insights into the Mechanism of AMP Resistance. J. Membr. Biol..

[60] Mhlongo J.T., Waddad A.Y., Albericio F., de la Torre B.G. (2023). Antimicrobial Peptide Synergies for Fighting Infectious Diseases. Adv. Sci..

[61] Zharkova M.S., Orlov D.S., Golubeva O.Y., Chakchir O.B., Eliseev I.E., Grinchuk T.M., Shamova O.V. (2019). Application of antimicrobial peptides of the innate immune system in combination with conventional antibiotics-a novel way to combat antibiotic resistance?. Front. Cell. Infect. Microbiol..

[62] European Committee for Antimicrobial Susceptibility Testing (EUCAST) of the European Society of Clinical Microbiology and Infectious Diseases (ESCMID) (2000). Terminology relating to methods for the determination of susceptibility of bacteria to antimicrobial agents. Clin. Microbiol. Infect. Off. Publ. Eur. Soc. Clin. Microbiol. Infect. Dis..

[63] Fratini F., Mancini S., Turchi B., Friscia E., Pistelli L., Giusti G., Cerri D. (2017). A novel interpretation of the Fractional Inhibitory Concentration Index: The case *Origanum vulgare* L. and *Leptospermum scoparium* J. R. et G. Forst essential oils against *Staphylococcus aureus* strains. Microbiol. Res..

[64] Witherell K.S., Price J., Bandaranayake A.D., Olson J., Call D.R. (2020). Circumventing colistin resistance by combining colistin and antimicrobial peptides to kill colistin-resistant and multidrug-resistant Gram-negative bacteria. J. Glob. Antimicrob. Resist..

[65] Sacco F., Bitossi C., Casciaro B., Loffredo M.R., Fabiano G., Torrini L., Raponi F., Raponi G., Mangoni M.L. (2022). The Antimicrobial Peptide Esc(1-21) Synergizes with Colistin in Inhibiting the Growth and in Killing Multidrug Resistant *Acinetobacter baumannii* Strains. Antibiotics.

[66] Taccetti G., Francalanci M., Pizzamiglio G., Messore B., Carnovale V., Cimino G., Cipolli M. (2021). Cystic Fibrosis: Recent Insights into Inhaled Antibiotic Treatment and Future Perspectives. Antibiotics.

[67] Hancock R.E.W., Alford M.A., Haney E.F. (2021). Antibiofilm activity of host defence peptides: Complexity provides opportunities. Nat. Rev. Microbiol..

[68] Di Somma A., Moretta A., Canè C., Cirillo A., Duilio A. (2020). Antimicrobial and antibiofilm peptides. Biomolecules.

[69] Ridyard K.E., Overhage J. (2021). The potential of human peptide ll-37 as an antimicrobial and anti-biofilm agent. Antibiotics.

[70] Siepi M., Oliva R., Masino A., Gaglione R., Arciello A., Russo R., Di Maro A., Zanfardino A., Varcamonti M., Petraccone L. (2021). Environment-sensitive fluorescent labelling of peptides by luciferin analogues. Int. J. Mol. Sci..

[71] Facchin B.M., dos Reis G.O., Vieira G.N., Mohr E.T.B., da Rosa J.S., Kretzer I.F., Demarchi I.G., Dalmarco E.M. (2022). Inflammatory biomarkers on an LPS-induced RAW 264.7 cell model: A systematic review and meta-analysis. Inflamm. Res..

[72] Torre A., Bacconi M., Sammicheli C., Galletti B., Laera D., Fontana M.R., Grandi G., De Gregorio E., Bagnoli F., Nuti S. (2015). Four-component *Staphylococcus aureus* vaccine 4C-staph enhances Fcγ receptor expression in neutrophils and monocytes and mitigates *S. aureus* infection in neutropenic mice. Infect. Immun..

[73] Baba T., Bae T., Schneewind O., Takeuchi F., Hiramatsu K. (2008). Genome sequence of *Staphylococcus aureus* strain newman and comparative analysis of staphylococcal genomes: Polymorphism and evolution of two major pathogenicity islands. J. Bacteriol..

[74] Zhorov B.S., Bregestovski P.D. (2000). Chloride channels of glycine and GABA receptors with blockers: Monte Carlo minimization and structure-activity relationships. Biophys. J..

[75] Siepi M., Morales-Narváez E., Domingo N., Monti D.M., Notomista E., Merkoçi A. (2017). Production of biofunctionalized MoS_2_ flakes with rationally modified lysozyme: A biocompatible 2D hybrid material. 2D Mater..

[76] Siepi M., Donadio G., Dardano P., De Stefano L., Monti D.M., Notomista E. (2019). Denatured lysozyme-coated carbon nanotubes: A versatile biohybrid material. Sci. Rep..

[77] Siepi M., Oliva R., Battista F., Petraccone L., Del Vecchio P., Izzo V., Dal Piaz F., Isticato R., Notomista E., Donadio G. (2020). Molecular dissection of dh3w, a fluorescent peptidyl sensor for zinc and mercury. Sensors.

[78] Wetlaufer D.B., Edsall J.T., Hollingworth B.R. (1958). Ultraviolet difference spectra of tyrosine groups in proteins and amino acids. J. Biol. Chem..

[79] Pane K., Verrillo M., Avitabile A., Pizzo E., Varcamonti M., Zanfardino A., Di Maro A., Rega C., Amoresano A., Izzo V. (2018). Chemical Cleavage of an Asp-Cys Sequence Allows Efficient Production of Recombinant Peptides with an N-Terminal Cysteine Residue. Bioconjug. Chem..

[80] Wiegand I., Hilpert K., Hancock R.E.W. (2008). Agar and broth dilution methods to determine the minimal inhibitory concentration (MIC) of antimicrobial substances. Nat. Protoc..

[81] Li J., Kleintschek T., Rieder A., Cheng Y., Baumbach T., Obst U., Schwartz T., Levkin P.A. (2013). Hydrophobic liquid-infused porous polymer surfaces for antibacterial applications. ACS Appl. Mater. Interfaces.

[82] Yu L., Tan M., Ho B., Ding J.L., Wohland T. (2006). Determination of critical micelle concentrations and aggregation numbers by fluorescence correlation spectroscopy: Aggregation of a lipopolysaccharide. Anal. Chim. Acta.

