# Supplementary material for: The Antimicrobial, Antibiofilm and Anti-Inflammatory Activities of P13#1, a Cathelicidin-like Achiral Peptoid
Source: Pharmaceuticals (Basel). 2023 Sep 30;16(10):1386. doi: 10.3390/ph16101386 (PMC10610514; doi:10.3390/ph16101386)
Supplement: Supplementary file 1 [file pharmaceuticals-16-01386-s001.zip › pharmaceuticals-2616808-supplementary.pdf]

The antimicrobial, antibiofilm and anti-inflammatory activities of P13#1, a cathelicidin-like achiral peptoid

Valeria Cafaro[1,†], Andrea Bosso[1,†], Ilaria Di Nardo[1,†], Assunta D'Amato[2], Irene Izzo[2], Francesco De Riccardis[2], Marialuisa Siepi[1], Rosanna Culurciello[1], Nunzia D'Urzo[1], Emiliano Chiarot[3], Antonina Torre[3], Elio Pizzo[1], Marcello Merola[1,*], Eugenio Notomista[1,*].

[1] Department of Biology, University of Naples Federico II, 80126 Naples, Italy
[2] Department of Chemistry and Biology "A. Zambelli", University of Salerno, 84084 Fisciano, Italy
[3] GSK, 53100 Siena, Italy
[†] Equally contributing authors.
* Authors to whom correspondence should be addressed (eugenio.notmista@unina.it, marcello.merola@unina.it)

Supplementary Information

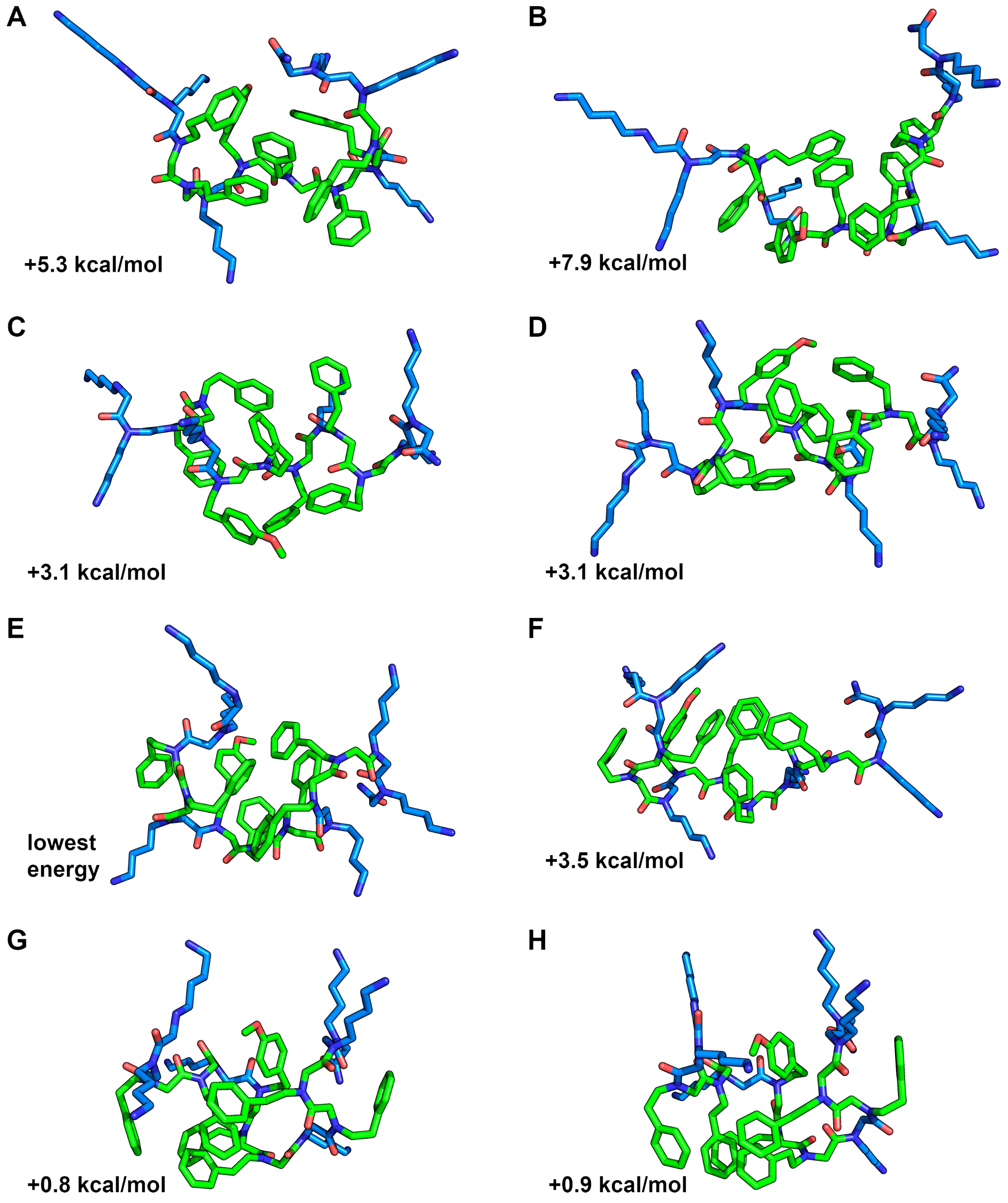

Fig. S1. Representative Monte-Carlo-minimized structures of P13#1 using water solvation. A-B) Structures with all-*trans* peptide bonds. C-D) Structures with all-*cis* peptide bonds. E-H) Structures with mixed *cis* and *trans* peptide bonds obtained by the random search described in Methods section 4.4. In each panel the number in lower left corner is the energy variation with respect to the lowest energy (that of structure in panel E). All the structures are oriented with the N-terminus on the left. Color code: oxygen, red; nitrogen, dark blue; carbon in hydrophobic residues, green; carbon in cationic residues, blue; carbon in prolines, grey.

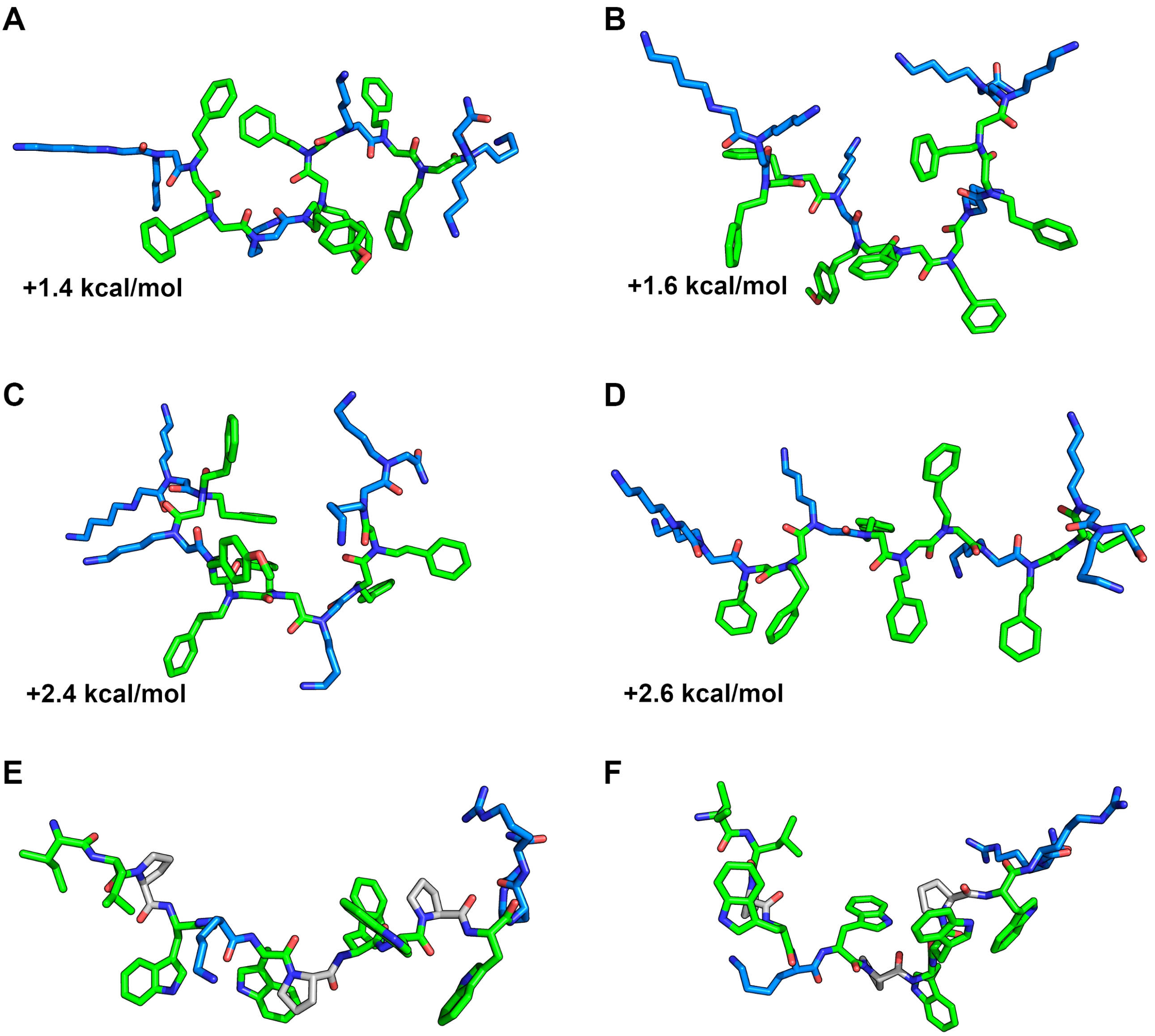

Fig. S2. Comparison between representative Monte-Carlo-minimized structures of P13#1 using octanol solvation and the NMR structures of bovine indolicidin. A-D) P13#1 structures with mixed *cis* and *trans* peptide bonds obtained by the random search described in Methods section 3.4. In each panel the number in lower left corner is the energy variation with respect to the lowest energy obtained using octanol solvation (all trans polyproline helix II-like conformation shown in panel A of Fig. 2). E-F) NMR structures of bovine indolicidin in SDS (PDB code: 1G8C) and dodecylphosphocholine (PDB code: 1G89), respectively. All the structures are oriented with the N-terminus on the left. Color code: oxygen, red; nitrogen, dark blue; carbon in hydrophobic residues, green; carbon in cationic residues, blue; carbon in prolines, grey.

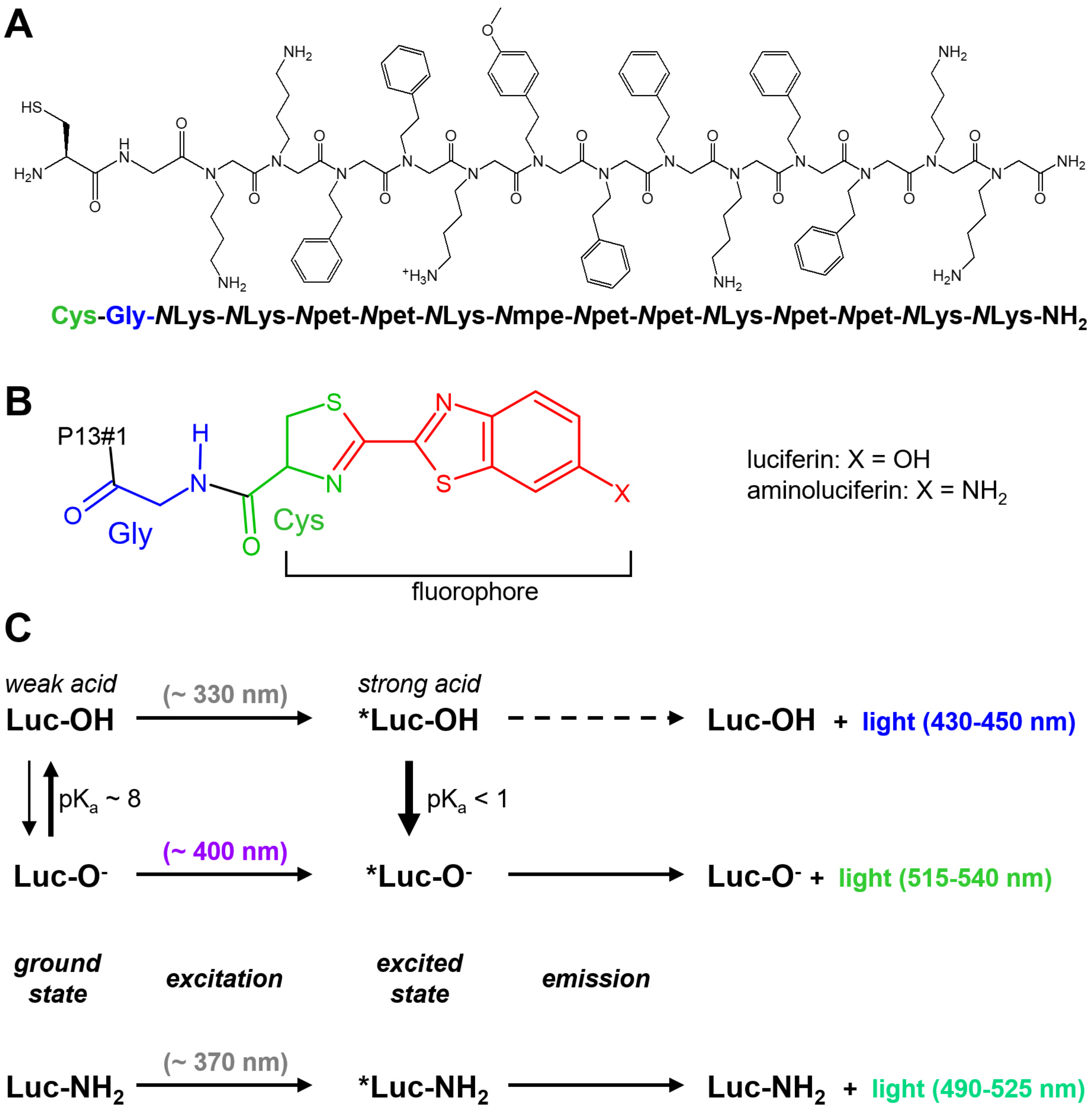

Fig. S3. Structure and fluorescence properties of the labeled peptoids and peptides. A) Structure of CG-P13#1. B) Schematic structure of Luc- and aLuc-P13#1. The additional Cys residues becomes an integral part of the fluorophore, whereas the Gly residues works as a spacer. C) Fluorescence properties of Luc- and aLuc-labeled species. Emissions shown on the right are common emission ranges, the actual emission wavelength is strongly dependent on environment polarity. The emission of neutral luciferin in the blue region (dashed line) can only be observed in the absence of any suitable proton acceptor for *Luc-OH.

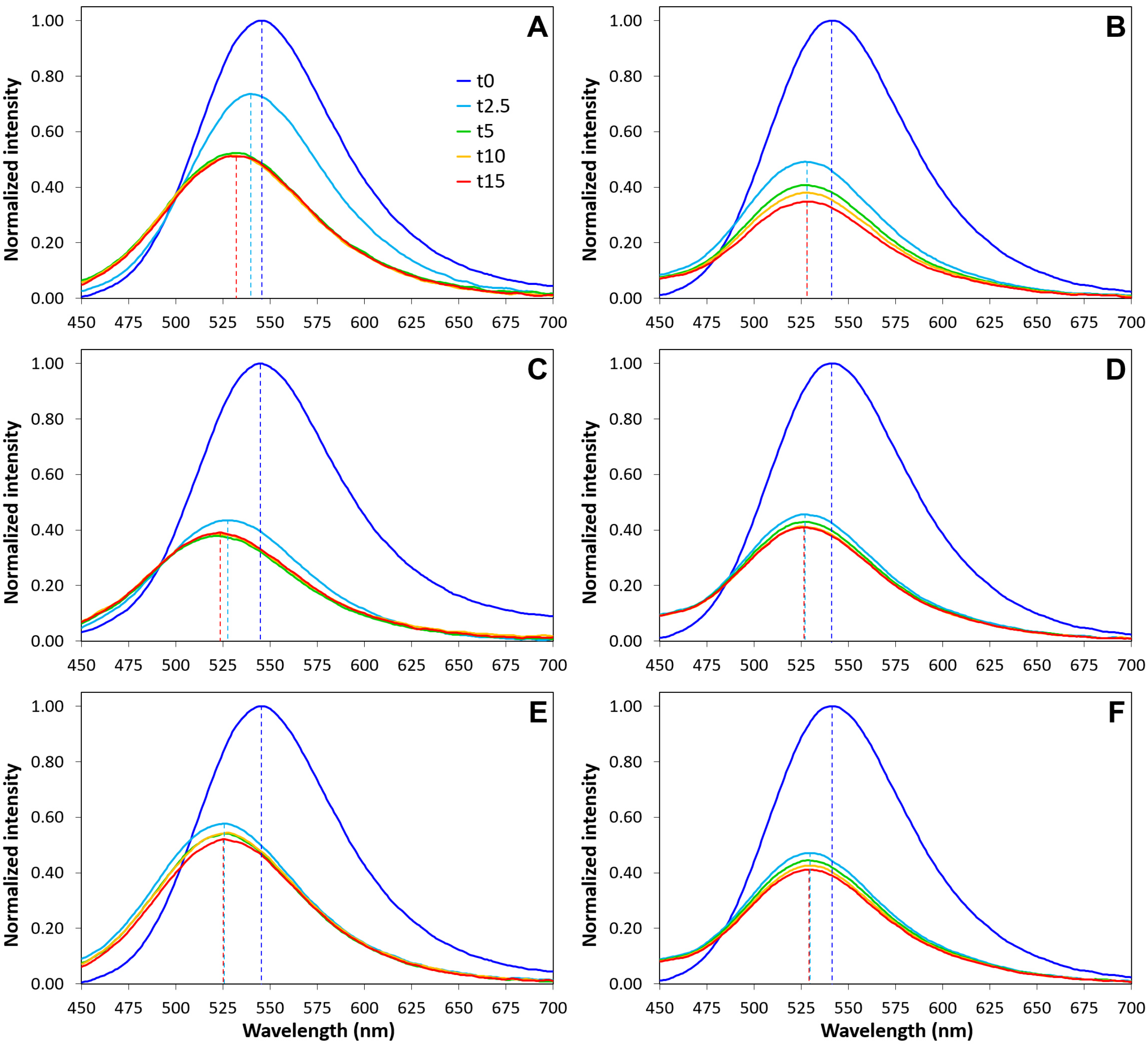

Fig. S4. Time variations in the emission spectra after excitation at 330 nm of Luc-GKY20 and Luc-P13#1 in the presence of bacterial cells. A, B) Emission spectra of Luc-GKY20 and Luc-P13#1, respectively, in the presence of *E. coli*. C, D) Emission spectra of Luc-GKY20 and Luc-P13#1, respectively, in the presence of *P. aeruginosa*. E, F) Emission spectra of Luc-GKY20 and Luc-P13#1, respectively, in the presence of *S. aureus*. In each panel the emission in the presence of bacterial cells was normalized to the emission before the addiction of cells.

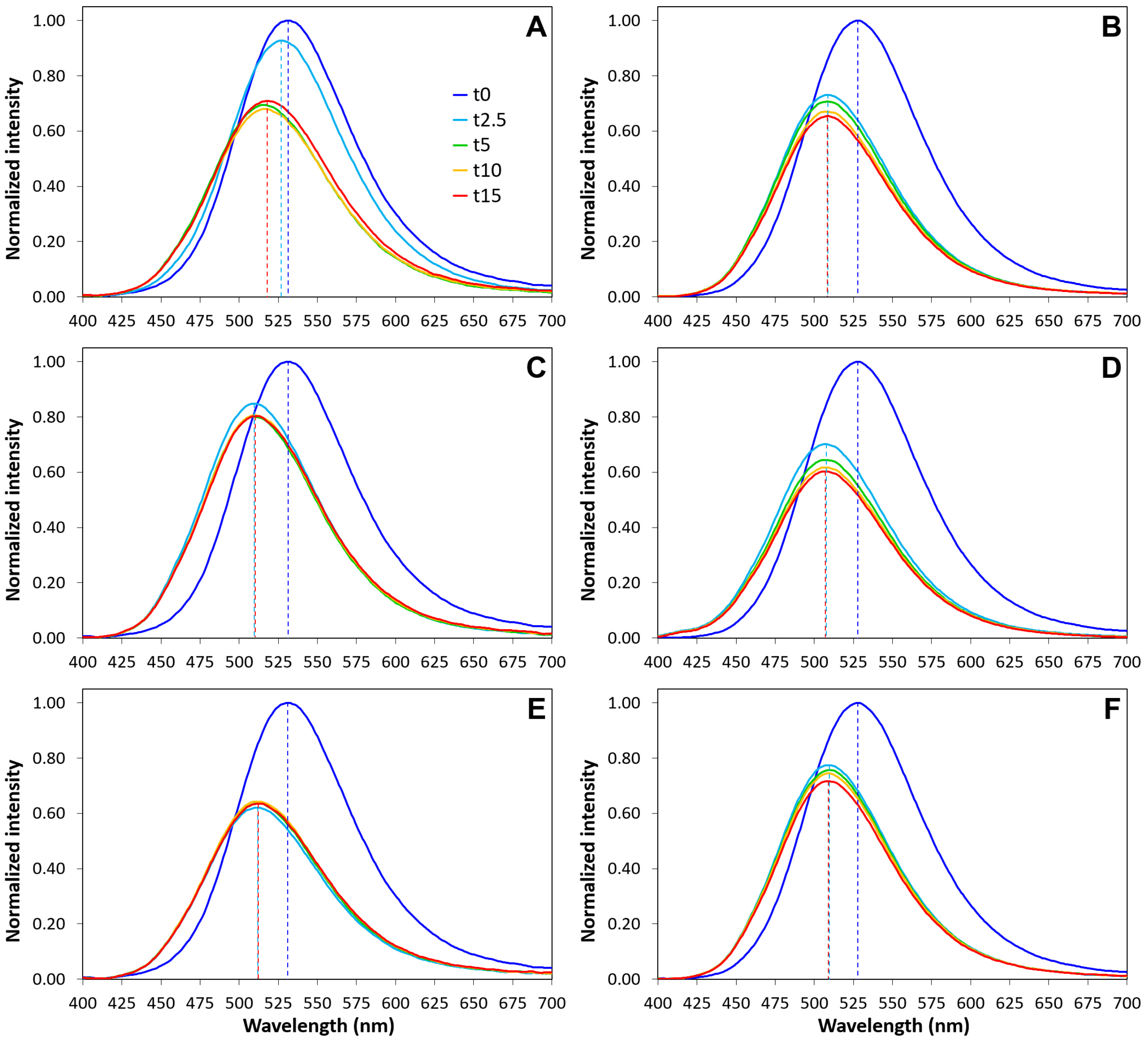

Fig. S5. Time variations in the emission spectra of aLuc-GKY20 and aLuc-P13#1 in the presence of bacterial cells. A, B) Emission spectra of Luc-GKY20 and Luc-P13#1, respectively, in the presence of *E. coli*. C, D) Emission spectra of Luc-GKY20 and Luc-P13#1, respectively, in the presence of *P. aeruginosa*. E, F) Emission spectra of Luc-GKY20 and Luc-P13#1, respectively, in the presence of *S. aureus*. In each panel the emission in the presence of bacterial cells was normalized to the emission before the addiction of cells.

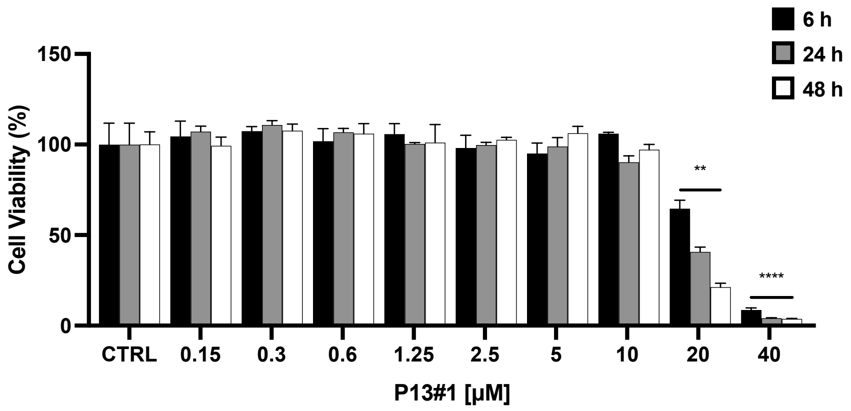

Fig. S6. Viability of Raw 264.7 murine macrophages cells treated with P13#1. Increasing concentrations of P13#1 (from 0.15 µM, corresponding to 0.34 µg/mL, to 40 µM, corresponding to 88 µg/mL) were administrated to cells for 6, 24 and 48 hours. Cell viability was determined by the MTT assay. Statistical analysis was carried out by GraphPad Prism using Student's t-test (* $p < 0.05$, ** $p < 0.01$, *** $p < 0.001$ and **** $p < 0.0001$).

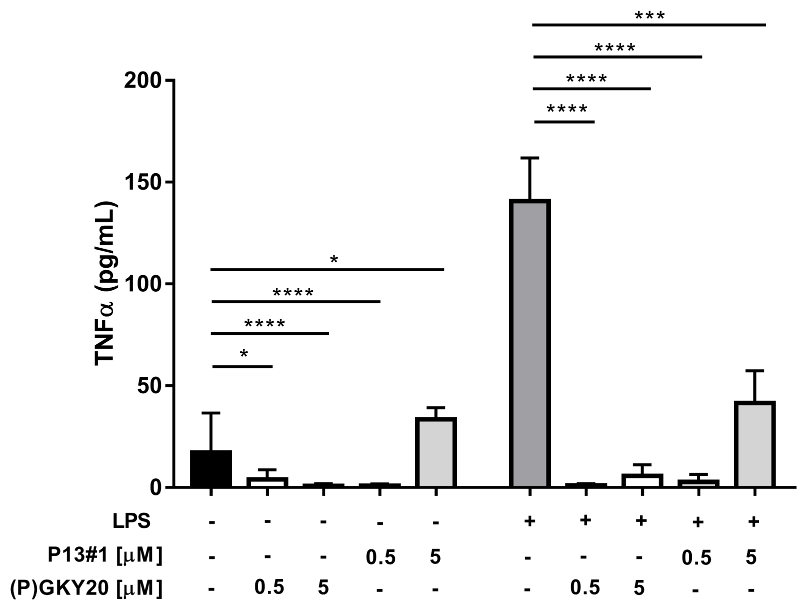

Fig. S7. Effects of (P)GKY20 and P13#1 on the release of TNFα from human THP-1 cells treated with LPS from *P. aeruginosa* 10. Statistical analysis was carried out by GraphPad Prism using Student's t-test (* $p < 0.05$, ** $p < 0.01$, *** $p < 0.001$ and **** $p < 0.0001$).

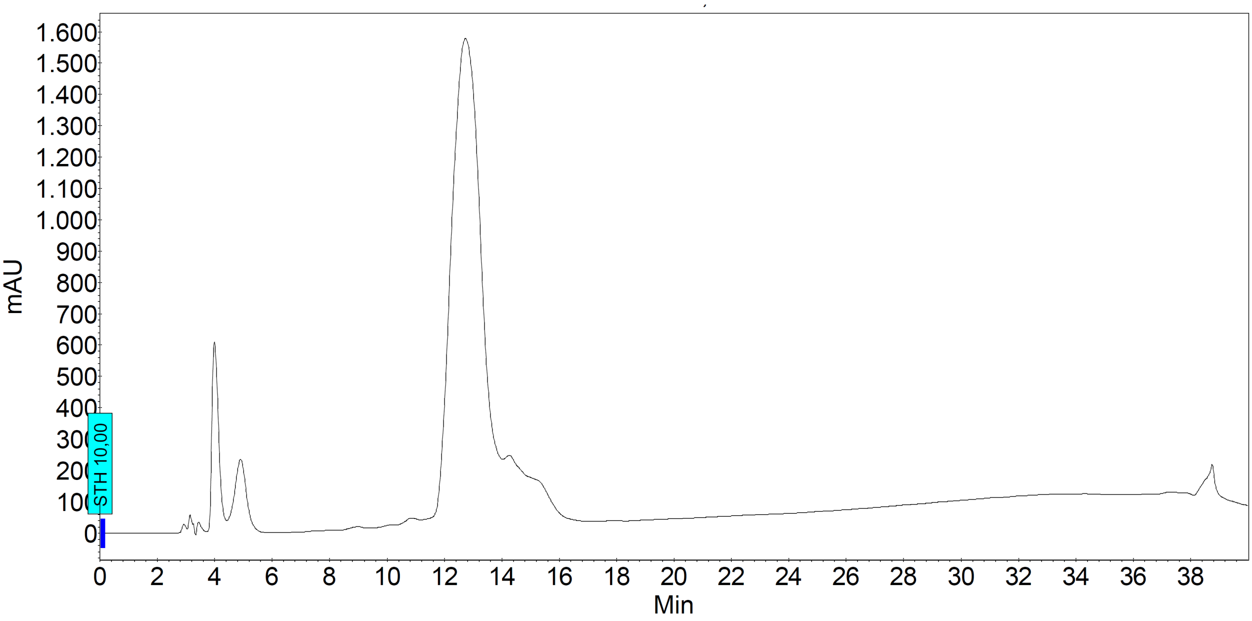

Fig. S8. **HPLC analysis of P13#1.** Conditions: 5 → 100% A in 30 min (A, 0.1% TFA in acetonitrile, B, 0.1% TFA in water); flow: 1 mL min^{-1}, 220 nm.

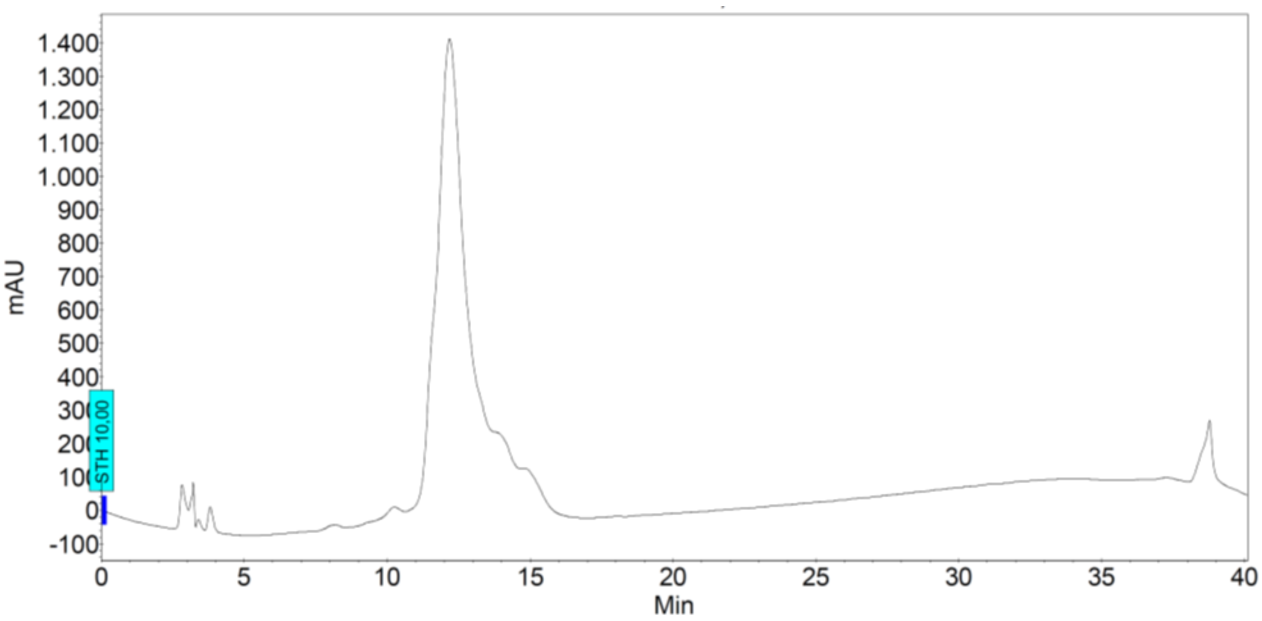

Fig. S9. **HPLC analysis of CG-P13#1.** Conditions: 5 → 100% A in 30 min (A, 0.1% TFA in acetonitrile, B, 0.1% TFA in water); flow: 1 mL min^{-1}, 220 nm.
